# Low-cost, open-source cell culture chamber for regulating physiologic oxygen levels

**DOI:** 10.1016/j.ohx.2021.e00253

**Published:** 2021-12-18

**Authors:** Colin R.N. Marchus, Jacob A. Knudson, Alexandra E. Morrison, Isabell K. Strawn, Andrew J. Hartman, Dev Shrestha, Nicholas M. Pancheri, Ian Glasgow, Nathan R. Schiele

**Affiliations:** a University of Idaho, Department of Chemical & Biological Engineering, Moscow, ID, United States; b University of Idaho, Department of Electrical and Computer Engineering, Moscow, ID, United States; c University of Idaho, Department of Mechanical Engineering, Moscow, ID, United States

**Keywords:** Hypoxia, Stem Cells, 3D printing, Control System

## Abstract

The physiological oxygen levels for several mammalian cell types *in vivo* are considered to be hypoxic (low oxygen tension), but the vast majority of *in vitro* mammalian cell culture is conducted at atmospheric oxygen levels of around 21%. In order to understand the impact of low oxygen environments on cells, oxygen levels need to be regulated during *in vitro* culture. Two common methods for simulating a hypoxic environment are through the regulation of gas composition or chemical induction. Chemically mimicking hypoxia can have adverse effects such as reducing cell viability, making oxygen regulation in cell culture chambers crucial for long-term culture. However, oxygen-regulating cell culture incubators and commercial hypoxia chambers may not always be a viable option due to cost and limited customization. Other low-cost chambers have been developed, but they tend to lack control systems or are fairly small scale. Thus, the objective of this project was to design and develop a low-cost, open-source, controllable, and reproducible hypoxia chamber that can fit inside a standard cell culture incubator. This design allows for the control of O_2_ between 1 and 21%, while maintaining CO_2_ levels at 5%, as well as monitoring of temperature, pressure, and relative humidity. Testing showed our hypoxia chamber was able to maintain CO_2_ levels at 5% and hypoxic O_2_ levels at 1% and 5% for long-term cell culture. This simple and easy-to-manufacture design uses off the shelf components, and the total material cost was $832.47 (USD).

## Graphical abstract

**Figure:**
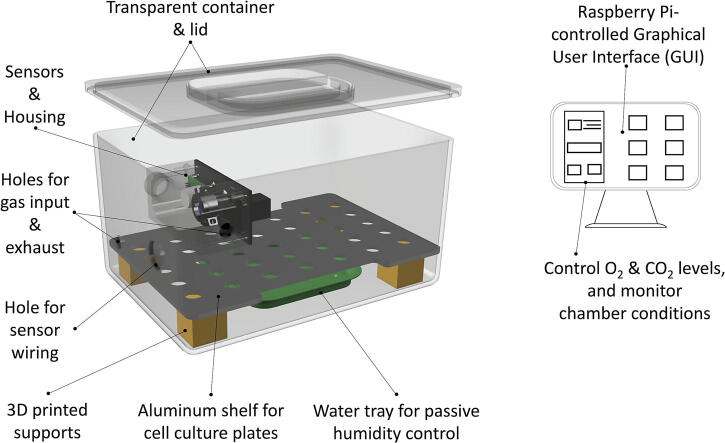

**Specifications table:** 

Hardware name	*Open-Source Hypoxia Chamber*
Subject area	• Pharmaceutical Science • Biological Sciences (e.g. Microbiology and Biochemistry)
Hardware type	• Biological sample handling and preparation
Open-Source License	GNU General Public License v3
Cost of Hardware	*∼$832.47 (USD)*
Source File Repository	https://doi.org/10.17605/OSF.IO/JVHDQ

## Hardware in context

Precise oxygenation is vital to mammalian tissue development and proper function [1]. Normal atmospheric oxygen (O_2_) levels (normoxia) are around 21%. Hypoxia, or a low level of oxygen, has been implicated in the pathogenesis of many debilitating conditions including various forms of cancer, chronic heart disease, pulmonary hypertension, and myocardial ischemia [2], [3], [4], [5], [6]. However, physiological oxygen levels in a number of tissues have also been measured to be hypoxic, with typical ranges in normal tissues from 1 to 14% oxygen [1], [7]. For example, the oxygen tension in articular cartilage appears to range from 1% to 10%, depending on the tissue depth [8]. In bone marrow, which contains a resident mesenchymal stem cell (MSC) population, oxygen levels have been estimated to fall between 1% and 7% [9], [10], [11]. While, these levels are considered hypoxic, compared to normal atmospheric conditions, these physiological oxygen levels play an important role in tissue maintenance and in regulation of stem cell differentiation [7].

Stem cells, such as those derived from bone marrow or adipose tissue, have been evaluated for use in tissue engineering and regenerative medicine therapies [12], [13], [14], [15]. Typical *in vitro* stem cell culture is conducted in a standard cell culture incubator at 37 °C, 5% carbon dioxide (CO_2_), and at atmospheric oxygen levels. However, based on the lower physiological oxygen levels, there is a need to explore how hypoxia impacts stem cell growth and differentiation. *In vitro* hypoxia has been shown to enhance the proliferation and viability of certain stem cells [16], [17], [18], [19], [20], [21], [22], [23], [24], [25], [26], [27]. For example, an oxygen concentration of 5% was found to increase tendon stem cell proliferation and reduce non-tendon gene expression (Sox9 and RunX2) [28]. In a different study, hypoxia may promote tenogenic differentiation of stem cells [29]. Taken together, exploring the effects of hypoxia on stem cell proliferation, differentiation, and self-renewal are needed to advance regenerative medicine. Furthermore, hypoxic conditions are needed to better understand disease pathogenesis in cells [30], [31], [32], and how oxygen concentration impacts tissue level mechanical properties [33]. Thus, there is a need to control oxygen levels during cell and tissue culture *in vitro*.

The primary techniques for mimicking a hypoxic environment include using chemical compounds that interact with hypoxia-induced signaling pathways in cells to mimic a hypoxic response, or by employing a hypoxia chamber to modulate oxygen gas levels (either through gaseous displacement using nitrogen (N_2_) or direct injection of O_2_). Chemically induced hypoxia aims to mimic the response of hypoxia inducible factor (HIF)-1, which is a transcription factor activated in cells by hypoxic environments [34]. However, commonly used HIF-1 chemical inducers (e.g., CoCl_2_) have been seen to reduce cell viability [35], [36], which may limit their use in long-term cell culture. Oxygen-regulating cell culture incubators and commercial hypoxia chambers offer an alternative to chemical hypoxia mimickers. However, these systems may be cost-prohibitive and are challenging to customize for specific laboratory needs. To address this, several low-cost hypoxia chamber systems have been previously developed [37], [38], [39]. These systems were designed with ease-of-use, portability, and low-cost materials in mind. However, these low-cost systems typically lack several important functions, such as controllable O_2_/CO_2_ levels, internal O_2_ and CO_2_ sensors to monitor and control levels, or a graphic user interface (GUI) for interacting with and controlling the system. One low-cost system did successfully implement a O_2_ sensor to track oxygen concentrations within a custom hypoxia chamber [40], but required the use of an anoxic gas mixture of 95% N_2_ and 5% CO_2_, and CO_2_ levels were not monitored or controlled. Further, many of the prior low-cost systems are small-scale and designed to accommodate one or two cell culture plates. There is a need for a low-cost hypoxia chamber that can fit inside a standard cell culture incubator, hold multiple standard cell culture plates, and include O_2_ and CO_2_ control and monitoring.

The goal of this project was to design and develop a novel low-cost, open-source, and easy to manufacture hypoxia chamber that can fit inside a standard cell culture incubator, hold cell culture flasks and well plates, and include O_2_ and CO_2_ control and monitoring (Specifications Table). Our system was designed to be readily replicated and easily modified, and maintain CO_2_ levels at 5%, while allowing the user to set O_2_ levels between 1% and 21%. In this system oxygen levels are controlled through gas displacement using N_2_, and a separate connection to a CO_2_ tank, reducing the need for pre-mixed gases. Gas displacement involves inputting a gas (e.g., N_2_) into the chamber, while having an exhaust port to displace the preexisting gas within the chamber, causing the overall O_2_ concentration to decrease [39]. Thus, this low-cost and easy to reproduce hypoxia chamber can be used to explore how physiological oxygen levels impact cells and tissues in culture to advance basic science as well as tissue engineering and regenerative medicine.

## Hardware description

**Fig. 1:**
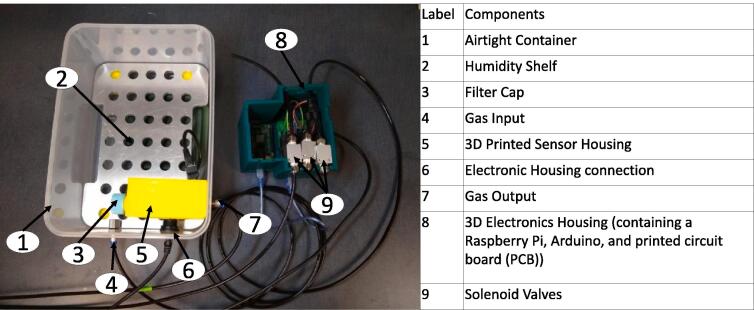
Hypoxia chamber.

The hypoxic chamber **(**Fig. 1**)** used in this design is a common household plastic storage container (i.e., a commercially purchased premade chamber), roughly 35.1 × 25.4 × 18.6 cm, that acts as an airtight chamber large enough to hold up to 10 standard cell culture plates (e.g., 96- to 6-well plates) or up to 5 standard cell culture flasks (e.g., T-75). Notably, the primary chamber can be unique to each future application, and requires only an airtight and modifiable (i.e., drill or machine holes of various sizes without sacrificing the structural integrity) container (in other words, it does not have to be identical to the container used in our assessments). The chamber (plastic storage container) is connected to rubber tubing that inputs either CO_2_ or N_2_ to achieve user-determined CO_2_ and O_2_ concentrations within the chamber **(**Fig. 1**)**. A second tube exhausts excess gas from the chamber to prevent pressurization, which is regulated by a solenoid valve. CO_2_ and N_2_ gas inflow are controlled via solenoids that open and close from a PID controller. The chamber internally contains a sensor housing unit, an aluminum shelf, shelf supports, and a water tray. The sensor housing unit (located in the path of the exhaust) contains the CO_2_ sensor (with integrated pressure and relative humidity sensors) and O_2_ sensor (with an integrated temperature sensor) and a filter to prevent potential contamination within the chamber. Similar to a cell culture incubator, the water tray maintains a relative humidity level of around 95%. To maintain temperature, the chamber is placed in a 37 °C cell culture incubator, or another user-specified temperature-controlled environment. The aluminum shelf containing multiple through-holes allows diffusion of water vapor throughout the chamber and holds the cell culture plates above the water tray. A separate external unit houses the electronics (PBC, Arduino, and Raspberry Pi) that controls the solenoids. These electronics are connected to the GUI, which is used by the operator to input and control the desired concentrations of O_2_ and CO_2_, as well as provide a real-time readout of O_2_ and CO_2_ concentrations, total pressure, temperature, and humidity within the chamber. Design highlights include:

• Varying container size (flexible design) to hold standard cell culture plates and flasks
• Fits into a standard cell culture incubator (interior incubator dimensions of 57.6 cm × 47 cm × 60.7 cm) and maintains 95% humidity
• Maintains 5% CO_2_
• O_2_ levels controlled and maintained between 1% and 21%
• Tunable gas inflow and exhaust rates
• Low cost (<$850 (USD), see Bill of Materials)
• Easy maintenance/sterilization using 70% ethanol
• Intuitive GUI

**Fig. 2:**
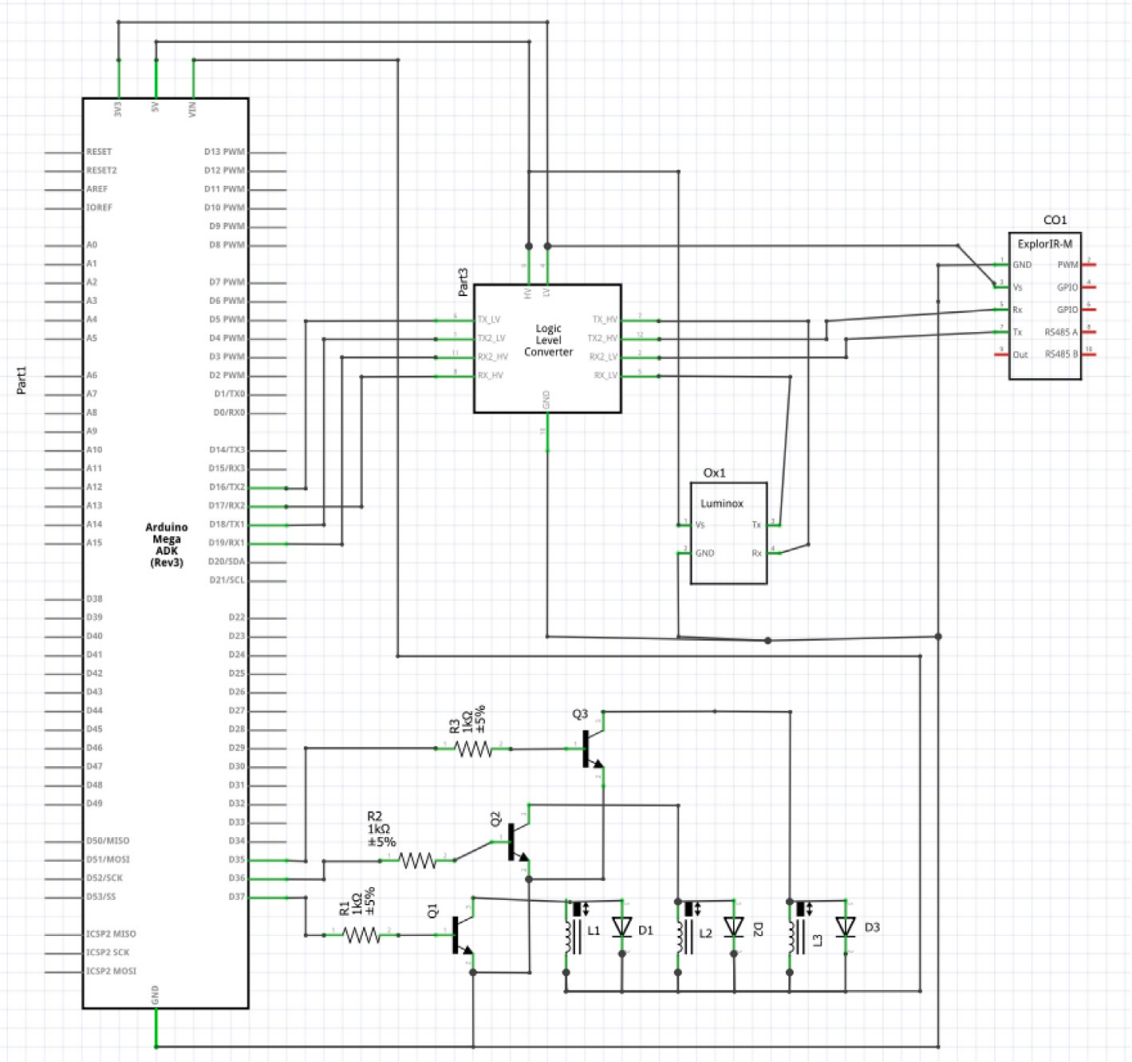
Overall electronic system schematic.

## Design files

The design files include three-dimensional (3D) drawings of all components as STL and Autodesk® Fusion 360 files (F3Z and F3D), as well as printed circuit board (PCB) design files, code for the controllers and GUI as outlined and described below and in the Design Files Summary.

### 3D printed parts

The aluminum shelf supports, sensor housing, electronic housing, and electronic housing lid were all 3D printed using Sindoh 3DWOX PLA filament. We recommend at least a 20% infill for all 3D printed parts, a printing temperature of at least 185 °C, and a layer height ranging between 0.2 and 0.3 mm (follow specific printer and material recommendations).

### 3D modeled parts

The modeled parts were used mainly for visualization purposes, using a 3D computer aided drafting (CAD) assembly. The parts that were modeled include the premade chamber, premade chamber lid, CO_2_ sensor, O_2_ sensor, MXboard, cell plate (well plate), aluminum shelf, aluminum shelf supports, water tray, sensor housing, RJ45 pinout, full design of electronic housing (including lid) and all pieces of the custom chamber design.

### Machined parts

The shelf was machined out of ¼” ± 0.125” aluminum sheet metal.

### Electronics

The overall schematic of the system is shown below in Fig. 2. It includes an Arduino Mega, the O_2_ and CO_2_ sensors, a logic level converter to ensure the 3.3 V UART signals of the sensors can communicate with the 5 V Arduino signals, three solenoids, 1kΩ resistor, BJT transistor, and a diode for each of the solenoids. The resistor-transistor-diode combination helps ensure that the 5 V Arduino signals will communicate properly with the 12 V solenoids, and that current spikes will not run back through the system when the solenoids close. The Arduino Mega was chosen due to both sensors needing UART serial communication ports. A Raspberry Pi, not pictured, was also used to help with data logging and to run the GUI.

### Software

There are two software sets that run the project. The Arduino has two files written in the default Arduino IDE that get compiled together to run and control the Arduino. These files are HypoxiaChamber.ino and ControlFunctions.ino. The Raspberry Pi has a python file that runs the GUI. GUI.py was created using the tkinter library in Python3.

**Design Files Summary:** 

Design file name	File type	Open source license	Location of the file
**Premade Chamber**			
Premade Chamber Full Design	STL	GNU General Public License v3	https://osf.io/pcxvw/
Premade Chamber Lid	STL	GNU General Public License v3	https://osf.io/95hms/
Pre-Made Chamber Shelf	STL	GNU General Public License v3	https://osf.io/b9s45/
Humidity_control_support	STL	GNU General Public License v3	https://osf.io/nymx2/
Cell Plate	STL	GNU General Public License v3	https://osf.io/xvays/
Water Tray	STL	GNU General Public License v3	https://osf.io/fuv7x/
Premade Chamber Full Design	F3Z	GNU General Public License v3	https://osf.io/h2yfw/
Premade Chamber Lid	F3D	GNU General Public License v3	https://osf.io/gnqz4/
Pre-Made Chamber Shelf	F3D	GNU General Public License v3	https://osf.io/3qvd6/
Humidity_control_support	F3D	GNU General Public License v3	https://osf.io/fd7nq/
Cell Plate	F3D	GNU General Public License v3	https://osf.io/4bq92/
Water Tray	F3D	GNU General Public License v3	https://osf.io/u4hvp/

**Sensor Housing**			
Housing and Sensors	STL	GNU General Public License v3	https://osf.io/wxp3z/
Sensor Housing Bottom	STL	GNU General Public License v3	https://osf.io/wksnp/
Sensor Housing Top	STL	GNU General Public License v3	https://osf.io/gcr4n/
CO2Sensor	STL	GNU General Public License v3	https://osf.io/fbuv6/
Luminox O2	STL	GNU General Public License v3	https://osf.io/r3jmf/
MXBoard	STL	GNU General Public License v3	https://osf.io/gdyqt/
O2Sensor Retainer	STL	GNU General Public License v3	https://osf.io/ymc6j/
RJ45 Pinout	STL	GNU General Public License v3	https://osf.io/z5fq9/
Housing and Sensors	F3Z	GNU General Public License v3	https://osf.io/563sc/
Sensor Housing Bottom	F3D	GNU General Public License v3	https://osf.io/e8utj/
Sensor Housing Top	F3D	GNU General Public License v3	https://osf.io/rj2u8/
CO2Sensor	F3D	GNU General Public License v3	https://osf.io/a92mx/
Luminox O2	F3D	GNU General Public License v3	https://osf.io/q3jvt/
MXBoard	F3D	GNU General Public License v3	https://osf.io/6h9g7/
O2Sensor Retainer	F3D	GNU General Public License v3	https://osf.io/78zrp/
RJ45 Pinout	F3D	GNU General Public License v3	https://osf.io/mzuqp/

**Electronic Housing**			
Electronic Housing and Lid	STL	GNU General Public License v3	https://osf.io/6re72/
Electronic Housing	STL	GNU General Public License v3	https://osf.io/cr9x2/
Electronic Housing Lid	STL	GNU General Public License v3	https://osf.io/93qth/
Electronic Housing and Lid	F3Z	GNU General Public License v3	https://osf.io/5ynbj/
Electronic Housing	F3D	GNU General Public License v3	https://osf.io/9yhpt/
Electronic Housing Lid	F3D	GNU General Public License v3	https://osf.io/ch2rp/

**PCB**			
Hypoxia_PCB	Brd	GNU General Public License v3	https://osf.io/q4stb/
Hypoxia_PCB	Sch	GNU General Public License v3	https://osf.io/nbguc/
Gerber Files	Zip	GNU General Public License v3	https://osf.io/54h87/

**Code**			
HypoxiaChamber	ino	GNU General Public License v3	https://osf.io/dm79y/
ControlFunctions	ino	GNU General Public License v3	https://osf.io/5a94y/
GUI	py	GNU General Public License v3	https://osf.io/er7cw/
settings	csv	GNU General Public License v3	https://osf.io/vwqc2/

**Gas Flow Validation**			
Premade Chamber Gas Flow Validation Folder	zip	GNU General Public License v3	https://osf.io/f9vy6/

**Custom Chamber**			
Custom Hypoxia Chamber	STL	GNU General Public License v3	https://osf.io/h7ncv/
Corner Piece Backside	STL	GNU General Public License v3	https://osf.io/pj28c/
Corner Piece For Frontside	STL	GNU General Public License v3	https://osf.io/2ckjp/
Custom Chamber Front Door Frame	STL	GNU General Public License v3	https://osf.io/7gns6/
Custom Chamber Front Door Removable	STL	GNU General Public License v3	https://osf.io/t5g6m/
Custom Chamber Left Sheet	STL	GNU General Public License v3	https://osf.io/5vy2f/
Custom Chamber Right Sheet	STL	GNU General Public License v3	https://osf.io/hdq3y/
Custom Hypoxia Chamber Humidity Shelf Supports	STL	GNU General Public License v3	https://osf.io/f5jyw/
Custom Hypoxia Chamber Humidity Shelf	STL	GNU General Public License v3	https://osf.io/qrgva/
Custom Chamber Top Sheet	STL	GNU General Public License v3	https://osf.io/pgsb6/
Custom Chamber Back Side	STL	GNU General Public License v3	https://osf.io/328a6/
Custom Chamber Bottom Sheet	STL	GNU General Public License v3	https://osf.io/sq4hf/
Custom Hypoxia Chamber	F3Z	GNU General Public License v3	https://osf.io/tu8b4/
Corner Piece Backside	F3D	GNU General Public License v3	https://osf.io/fv423/
Corner Piece For Frontside	F3D	GNU General Public License v3	https://osf.io/4q7d5/
Custom Chamber Front Door Frame	F3D	GNU General Public License v3	https://osf.io/qbuk6/
Custom Chamber Front Door Removable	F3D	GNU General Public License v3	https://osf.io/pu7je/
Custom Chamber Left Sheet	F3D	GNU General Public License v3	https://osf.io/fvjuq/
Custom Chamber Right Sheet	F3D	GNU General Public License v3	https://osf.io/y6c72/
Custom Hypoxia Chamber Humidity Shelf Supports	F3D	GNU General Public License v3	https://osf.io/9yt2j/
Custom Hypoxia Chamber Humidity Shelf	F3D	GNU General Public License v3	https://osf.io/3ezqa/
Custom Chamber Top Sheet	F3D	GNU General Public License v3	https://osf.io/7z8sg/
Custom Chamber Back Side	F3D	GNU General Public License v3	https://osf.io/tpz5n/
Custom Chamber Bottom Sheet	F3D	GNU General Public License v3	https://osf.io/r6dqn/

Premade Chamber:

**Premade Chamber Full Design**: This is a model of the chamber with all other components placed inside. It was used for visualization purposes as well as determining exhaust, input and ethernet connector hole locations. Thus, it may not include all details to exact specifications as found in the final design build and is herein referred to as a prototype model.

**Premade Chamber Lid**: This is a model of the lid for the airtight plastic container. This is a prototype model that was made solely for visualization purposes.

**Pre-Made Chamber Shelf**: This file is for the design of the aluminum shelf which supports the cell plates.

**Humidity_control_support**: This is the file for the 3D printed supports which are used to support the shelf in the bottom of the chamber.

**Cell Plate**: This is a model of one of the cell plates that can be used inside of the chamber. This is a prototype model that was made solely for visualization purposes.

**Water Tray**: This is a model of the water tray used for maintaining humidity within the chamber. This is a prototype model that was made solely for visualization purposes.

Sensor Housing:

**Housing and Sensors:** This is a prototype model of the sensor housing unit. This model was used to visualize the configuration of each of the sensors and the RJ45 Pinout.

**Sensor Housing Bottom:** 3D printed bottom piece of the sensor housing unit.

**Sensor Housing Top:** 3D printed top piece of the sensor housing unit.

**CO_2_ Sensor**: This is a model of the CO_2_ sensor. This is a prototype model that was made solely for visualization purposes.

**Luminox O2**: This is a model of the O_2_ sensor. This is a prototype model that was made solely for visualization purposes.

**MXBoard**: This is a model of the MXBoard that the CO_2_ sensor is connected to. This is a prototype model that was made solely for visualization purposes.

**O2Sensor Retainer**: This file is for a 3D printed piece which holds the O_2_ sensor in place in the sensor housing.

**RJ45 Pinout**: This is a model of the RJ45 pinout which allows the sensors inside the sensor housing to be connected via ethernet cable (in this paper this part is also referred to as an ethernet connector). This is a prototype model that was made solely for visualization purposes.

Electronic Housing:

**Electronic Housing and Lid:** This is a model of the electronic housing, lid, Raspberry Pi, PCB, Solenoids, and Arduino. This model was used to represent the configuration of all of these parts together. The Raspberry Pi, PCB, Solenoids, and Arduino are prototype models used for visualization purposes.

**Electronic Housing:** 3D printed housing unit used to house the Raspberry Pi, PCB, Solenoids, and Arduino.

**Electronic Housing Lid:** 3D printed lid used to cover the top portion of the electronic housing unit.

PCB:

**Hypoxia_PCB:** These are the schematic (.sch) and board (.brd) views created in Autodesk Eagle software to create the PCB used in this project.

**GerberFiles:** This zipped folder contains the files required for manufacturing the PCB designed for this project. Simply send this zipped folder to the manufacturer to have the board created as is.

Code:

**HypoxiaChamber:** This is the main Arduino code to control the system. Contains the setup and main loop of the program. It requires the installation of the Arduino PID library by Brett Beauregard. The following link goes to the GitHub related to this library: https://github.com/br3ttb/Arduino-PID-Library

**ControlFunctions:** This is the auxiliary file for the Arduino code, it contains the command parsing functions, O_2_ and CO_2_ solenoid control function, and O_2_ and CO_2_ read functions.

**GUI:** This is the python3 file to run on the Raspberry Pi which runs the user interface. It is built using the tkinter UI library. The required python libraries are the following: tkinter, serial, time, csv, datetime, shutil.

**settings:** This is the comma separated values (CSV) file that the GUI uses to store the default settings of the GUI. Place it in the same directory as the GUI.py file before starting the GUI.

*Custom Chamber:

**Custom Hypoxia Chamber:** This is a model of the custom chamber that was designed, but never fully implemented. It was included here for users that would prefer a custom-build chamber.

**Corner Piece Backside:** 3D model of the 4 corner pieces that were used on the back side of the custom chamber.

**Corner Piece For Frontside:** 3D model of the 4 corner pieces that were used on the front side of the custom chamber.

**Custom Chamber Front Door Frame:** 3D model of the frame of the door.

**Custom Chamber Front Door Removable:** 3D model of the removable portion of the door.

**Custom Chamber Left Sheet:** 3D model of the left sheet of the custom chamber.

**Custom Chamber Right Sheet:** 3D model of the right sheet of the custom chamber.

**Custom Chamber Shelf Supports:** 3D model of the shelf supports for the humidity shelf for the custom chamber.

**Custom Chamber Shelf:** 3D model of the humidity shelf for the custom chamber

**Custom Chamber Top Sheet:** 3D model of the top sheet of the custom chamber.

**Custom Chamber Back Side:** 3D model of the back sheet of the custom chamber.

**Custom Chamber Bottom Sheet:** 3D model of the bottom sheet of the custom chamber.

*This custom chamber design has not been fully tested or constructed.

## Bill of materials

**Bill of Materials:** 

Part reference	Component	Quantity	Unit cost (USD)	Total cost (USD)	Link	Material type
**Control System & GUI**						
10pcs transistors w/ diodes	TIP120 NPN BJT ST Darlington Transistor	1	9.99	9.99	https://urldefense.com/v3/__https:/www.amazon.com/gp/r.html?C=1GDZONJ9HF37K&K=KJK9P5DVG8T1&M=urn:rtn:msg:202011051541537fc1eda1459f4ac0aa3fa33a9840p0na&R=242BUMLMXU7B4&T=C&U=http*3A*2F*2Fwww.amazon.com*2Fdp*2FB00FVLGYEY*2Fref*3Dpe_386300_440135490_TE_item&H=GQH4ETEFC1KFZHA1LV9ZLMZDPWMA&ref_=pe_386300_440135490_TE_item__;JSUlJSUlJQ!!JYXjzlvb!245fCaDZNDMG5UdG8kAz7chrJo3c61NjhhYEUMGPFn5A5vORH-B5lhMWlaWYpEAnAg$	Silicon, Germanium
SHCS 6–32 0.5″ 100 pack	Socket Head Screw 6–32 Thread Size, 1/2″ Long	1	9.59	9.59	https://www.mcmaster.com/91251A148/	Black-Oxide alloy steel
SHCS 6–32 0.75″ 100 pack	Socket Head Screw 6–32 Thread Size, 3/4″ Long	1	10.04	10.04	https://www.mcmaster.com/91251A151/	Black-oxide alloy steel
Hex Nut 6–32 100 pack	18–8 Stainless Steel Hex Nut 6–32 Thread	1	3.18	3.18	https://www.mcmaster.com/91841A007/	Stainless steel
SHCS M1.6 14 mm 25 pack	Steel Socket Head Screw M1.6 × 0.35 mm Thread, 14 mm Long	1	8.97	8.97	https://www.mcmaster.com/91292A311/	Stainless steel
Hex Nut M1.6 25 pack	Steel Hex Nut M1.6 × 0.35 mm Thread	1	9.19	9.19	https://www.mcmaster.com/91828A006/	Stainless steel
CO_2_ sensor	ExplorIR®-M 20% CO_2_ Sensor	1	179.00	179.00	https://www.co2meter.com/products/explorir-10-co2-smart-led-sensor?variant=20504457773174	Metal, polymer
Computer monitor	Sceptre 20″ 1600x900 75 Hz Ultra Thin LED Monitor	1	74.99	74.99	https://www.amazon.com/Sceptre-E205W-16003R-Frameless-Speakers-Metallic/dp/B0933GJB3V/ref=sr_1_3?dchild=1&keywords=pc%2Bmonitor&qid=1620096094&refinements=p_36%3A1253505011&rnid=386442011&s=electronics&sr=1-3&th=1	Liquid crystals, glass, plastic, polymer
Ethernet cable 2 pack	Cat6 Ethernet Cable, 15 Feet (2 Pack)	1	11.95	11.95	https://www.amazon.com/Ethernet-Cable-Meters-Network-Internet/dp/B00GBBSSCO?th=1	Metal
Ethernet connector	Jack Modular Connector 8p8c (RJ45, Ethernet) 90° Angle (Right) Shielded	1	2.71	2.71	https://www.digikey.com/en/products/detail/kycon,-inc/GLX-S-88M/9976001?utm_adgroup=General&utm_source=google&utm_medium=cpc&utm_campaign=Smart%20Shopping_Product_Zombie%20SKUS&utm_term=&utm_content=General&gclid=Cj0KCQiAyoeCBhCTARIsAOfpKxhjDYnKYlMoEdt9ZZidDeE-KhdyH3cyFHw4T3J6I7_so1gIMFggVXgaAmetEALw_wcB	PBT plastic
Keyboard & mouse	Macally USB Wireless Keyboard and Mouse Combo	1	21.99	21.99	https://www.amazon.com/Macally-Wireless-Keyboard-Mouse-Combo/dp/B08CHFKQN1/ref=sr_1_12_sspa?dchild=1&keywords=keyboard+and+mouse&qid=1620096535&refinements=p_36%3A1253503011&rnid=386442011&s=electronics&sr=1-12-spons&psc=1&spLa=ZW5jcnlwdGVkUXVhbGlmaWVyPUEzMlg4MkZaWTdCOFg1JmVuY3J5cHRlZElkPUEwMTc3OTcyMlJJMFNRWU1CWkdIMSZlbmNyeXB0ZWRBZElkPUEwOTM1NzkzMk5DOURWWVFLRTcxUCZ3aWRnZXROYW1lPXNwX210ZiZhY3Rpb249Y2xpY2tSZWRpcmVjdCZkb05vdExvZ0NsaWNrPXRydWU=	ABS plastic, rubber, metal
Logic level converter	BSS138 Logic-Level Translator Interface Evaluation Board	1	2.95	2.95	https://www.digikey.com/en/products/detail/sparkfun-electronics/BOB-12009/5673795?utm_adgroup=Evaluation%20and%20Demonstration%20Boards%20and%20Kits&utm_source=google&utm_medium=cpc&utm_campaign=Shopping_Product_Development%20Boards%2C%20Kits%2C%20Programmers_NEW&utm_term=&utm_content=Evaluation%20and%20Demonstration%20Boards%20and%20Kits&gclid=Cj0KCQiA48j9BRC-ARIsAMQu3WR4Temqnqc6yToA_0f6D6nzPL5FcZR4MZX3MH4v8D5-2UfYSYSLp44aAuTbEALw_wcB	Metal, polymer
Mega Microcontroller	ELEGOO MEGA 2560 R3 Board ATmega2560	1	15.86	15.86	https://urldefense.com/v3/__https:/www.amazon.com/gp/r.html?C=1GDZONJ9HF37K&K=KJK9P5DVG8T1&M=urn:rtn:msg:202011051541537fc1eda1459f4ac0aa3fa33a9840p0na&R=2AYRAWU7M3IAJ&T=C&U=http*3A*2F*2Fwww.amazon.com*2Fdp*2FB01H4ZLZLQ*2Fref*3Dpe_386300_440135490_TE_item&H=TDQ4LURSEYNYDOG00A86K8OSIO0A&ref_=pe_386300_440135490_TE_item__;JSUlJSUlJQ!!JYXjzlvb!245fCaDZNDMG5UdG8kAz7chrJo3c61NjhhYEUMGPFn5A5vORH-B5lhMWlaWCrEHEww$	Metal, polymer
Micro HDMI to HDMI cable	Micro HDMI to HDMI Adapter Cable, Wenter 6.5ft	1	6.99	6.99	https://www.amazon.com/Adapter-Wenter-Action-Camera-Supported/dp/B07VRCK5W1/ref=sr_1_4?dchild=1&keywords=micro%2Bhdmi%2Bto%2Bhdmi%2Bcable&qid=1617056683&s=electronics&sr=1-4&th=1	Metal, polymer
Oxygen sensor	LuminOX LOX-02 25% Oxygen Sensor	1	99.00	99.00	https://gaslab.com/products/oxygen-sensor-luminox-lox-o2	Metal, polymer
Panel mount ethernet cable	RJ-45 Ethernet Round Panel Mount Extension Cable − 30 cm	1	4.95	4.95	https://www.adafruit.com/product/4215?gclid=Cj0KCQiAtqL-BRC0ARIsAF4K3WEdhGpvej4_MeRI-yRW-DvLTbiypVoap2BQdFTcSuo7qlzrlPyxzO4aAs-uEALw_wcB	Metal, polymer
PCB	Printed circuit board	1	2.00	2.00	https://jlcpcb.com/	Epoxy, metal, Teflon
Power supply	DC 12 V 3A switching power supply	1	12.50	12.50	https://urldefense.com/v3/__https:/www.amazon.com/gp/r.html?C=1GDZONJ9HF37K&K=KJK9P5DVG8T1&M=urn:rtn:msg:202011051541537fc1eda1459f4ac0aa3fa33a9840p0na&R=22Z438R7AWD5O&T=C&U=http*3A*2F*2Fwww.amazon.com*2Fdp*2FB073WSWT34*2Fref*3Dpe_386300_440135490_TE_item&H=MERMXVYUMAKDBKOTONS1NRUQPYGA&ref_=pe_386300_440135490_TE_item__;JSUlJSUlJQ!!JYXjzlvb!245fCaDZNDMG5UdG8kAz7chrJo3c61NjhhYEUMGPFn5A5vORH-B5lhMWlaXPGkPprA$	Semi-conductor, metal
Raspberry pi 4	Raspberry Pi 4 Model B − 2 GB RAM	1	35.00	35.00	https://www.adafruit.com/product/4292	Metal, polymer
Raspberry pi power supply	Official Raspberry Pi Power Supply 5.1 V 3A with USB C − 1.5 m long	1	7.95	7.95	https://www.adafruit.com/product/4298?gclid=Cj0KCQiAnKeCBhDPARIsAFDTLTJ5xF08gAsjRCXjiWwyCOixsO993ms8-xlPY8BIEjogagroAUF8YU4aAhiQEALw_wcB	Semi-conductor, metal
RJ45 terminal adapter connecter	2pack RJ45 /8p8c Female Jack to 8 Pin Screw Terminal Connector	1	9.59	9.59	https://www.amazon.com/Poyiccot-Compatible-Terminal-Connector-Ethernet/dp/B07WKKVZRF/ref=pd_lpo_328_t_0/146-1998248-4050046?_encoding=UTF8&pd_rd_i=B07WKKVZRF&pd_rd_r=bc8784f0-33f7-4bbe-b64c-f0603cc0c1e1&pd_rd_w=iAZNO&pd_rd_wg=Zy6MQ&pf_rd_p=7b36d496-f366-4631-94d3-61b87b52511b&pf_rd_r=KZWXS2H88PR4PJQMA02Z&psc=1&refRID=KZWXS2H88PR4PJQMA02Z	Metal, polymer
SD card	Samsung (MB-ME32GA/AM) 32 GB 95 MB/s (U1) microSDHC EVO Select Memory Card with Full-Size Adapter	1	7.99	7.99	https://www.amazon.com/Samsung-MicroSDHC-Adapter-MB-ME32GA-AM/dp/B06XWN9Q99/ref=pd_bxgy_3/144-4258773-7962047?_encoding=UTF8&pd_rd_i=B06XWN9Q99&pd_rd_r=62af970e-e8a2-4a75-9650-83cca9029fc4&pd_rd_w=pgsqV&pd_rd_wg=KkUXY&pf_rd_p=ce6c479b-ef53-49a6-845b-bbbf35c28dd3&pf_rd_r=HN8H466W1DE9M42BN6FB&psc=1&refRID=HN8H466W1DE9M42BN6FB	PVC plastic, metal
Solenoids	Tailonz Pneumatic 1/4 in. NPT 12 V	3	11.99	35.97	https://urldefense.com/v3/__https:/www.amazon.com/gp/r.html?C=1GDZONJ9HF37K&K=KJK9P5DVG8T1&M=urn:rtn:msg:202011051541537fc1eda1459f4ac0aa3fa33a9840p0na&R=2ZKPJ4FHCMIVV&T=C&U=http*3A*2F*2Fwww.amazon.com*2Fdp*2FB07XB6LF49*2Fref*3Dpe_386300_440135490_TE_item&H=YHMO0AVCZZN5OWPHOLZZDLWWMOSA&ref_=pe_386300_440135490_TE_item__;JSUlJSUlJQ!!JYXjzlvb!245fCaDZNDMG5UdG8kAz7chrJo3c61NjhhYEUMGPFn5A5vORH-B5lhMWlaUagU0A4Q$	Metal, plastic
Wires	Premium Female/Male 'Extension' Jumper Wires − 20 × 3″	1	1.95	1.95	https://www.adafruit.com/product/1953?gclid=Cj0KCQjw4cOEBhDMARIsAA3XDRjSq1_YMg2-skHVB9QnPDxH9WkLnwmsFdBjMxNzXkja5uUA2reROjYaAm9hEALw_wcB	Metal, rubber
**Chamber, Gas & Tubing components**						
3D printer filament	3D printer yellow PLA filament spool, 1.75 mm diameter	1	25.50	25.50	https://urldefense.com/v3/__https:/www.amazon.com/gp/r.html?C=1GDZONJ9HF37K&K=KJK9P5DVG8T1&M=urn:rtn:msg:202011051541537fc1eda1459f4ac0aa3fa33a9840p0na&R=24JZMX5HUFGGS&T=C&U=http*3A*2F*2Fwww.amazon.com*2Fdp*2FB01HD9AIJI*2Fref*3Dpe_386300_440135490_TE_item&H=HV3XYBDD01XSJKZCPB5A7UCQ5MUA&ref_=pe_386300_440135490_TE_item__;JSUlJSUlJQ!!JYXjzlvb!245fCaDZNDMG5UdG8kAz7chrJo3c61NjhhYEUMGPFn5A5vORH-B5lhMWlaWTa1ku4A$	PLA
5 pack heavy duty water tray	5 Pack Green Plant Saucer Heavy Duty Sturdy Drip Trays	1	9.99	9.99	https://urldefense.com/v3/__https:/www.amazon.com/gp/r.html?C=1GDZONJ9HF37K&K=KJK9P5DVG8T1&M=urn:rtn:msg:20210205000523ea0578565313430fae02050b0270p0na&R=1V289JBVO9DH7&T=C&U=http*3A*2F*2Fwww.amazon.com*2Fdp*2FB08LSTQ9DW*2Fref*3Dpe_386300_440135490_TE_item&H=F38QYKGFIFHU5ATP6FATHIDFBG4A&ref_=pe_386300_440135490_TE_item__;JSUlJSUlJQ!!JYXjzlvb!1ZIUksOaqpmS53XGL69klMhWQJkBvUSPZNJFENLWoJdMum3f5rip4kzHPUfIO5k9cw$	PVC
Airtight container	Komax Biokips Extra Large Food Storage Container	1	21.99	21.99	https://urldefense.com/v3/__https:/www.amazon.com/gp/r.html?C=1GDZONJ9HF37K&K=KJK9P5DVG8T1&M=urn:rtn:msg:202011051541537fc1eda1459f4ac0aa3fa33a9840p0na&R=2I0U3J9WZ428H&T=C&U=http*3A*2F*2Fwww.amazon.com*2Fdp*2FB00NMRDQRM*2Fref*3Dpe_386300_440135490_TE_item&H=4MMIQBWLWZOFGLLCJPBZDXTGBXGA&ref_=pe_386300_440135490_TE_item__;JSUlJSUlJQ!!JYXjzlvb!245fCaDZNDMG5UdG8kAz7chrJo3c61NjhhYEUMGPFn5A5vORH-B5lhMWlaVjhXvMZQ$	Plastic
Aluminum	0.375″ Aluminum Plate	1	35.00	35.00	https://www.onlinemetals.com/en/buy/aluminum/-special-buy-0-375-aluminum-plate-6061-t651/pid/23816	Aluminum
Barbed tube fitting	Plastic Barbed Tube Fitting for Air and Water Tight-Seal, Connector, for 5/32″ Tube ID	1	8.67	8.67	https://www.mcmaster.com/5463K338-2974K221/	Nylon plastic
CO_2_ tank	Compressed CO_2_ tank	1	55.84	55.84	http://www.oxarc.com/	Carbon Dioxide
Filter cap	Vented, Sterile Caps for 25 mL & 50 mL Suspension Flasks, pack/5	1	26.30	26.30	https://www.calpaclab.com/vented-sterile-caps-for-25ml-50ml-suspension-flasks-chemglass/cg-cls-2410-v01?utm_source=googleshopping&gclid=Cj0KCQjwtsv7BRCmARIsANu-CQdxlr9C_Fzq84GZltD6h7Xi2g7sv_qhi7-BsHwY6I1O1thvnAroZ1waAr03EALw_wcB	Polypropylene
Nitrogen tank	Compressed nitrogen tank	1	34.28	34.28	http://www.oxarc.com/	Nitrogen
Push to connect wye connecter	Push-to-Connect Tube Fitting for Air Wye Connector, for 6 mm Tube OD	1	5.30	5.30	https://www.mcmaster.com/5225K87/	Nylon plastic
Rubber tubing	Firm Polyurethane Rubber Tubing for Air and Water 4 mm ID, 6 mm OD	1	10.00	10.00	https://www.mcmaster.com/50315K24/	Polyurethane rubber
Thru wall gas adapter	Thru-Wall Adapter, for 6 mm Tube OD × 1/4 BSPT Female	2	7.65	15.30	https://www.mcmaster.com/1201N92/	Metal, nylon plastic, polyurethane rubber
**Total Price of Chamber and Components: $832.47 (USD)**						

## Build instructions

### Premade chamber construction

• 5.1.1) 1x Outer Chamber
  o A) Drill 3 holes into the chamber: gas input hole, gas exhaust hole, and the hole for the ethernet cable (reference *Premade Chamber Full Design* design file for specific hole location) **(**Fig. 4 **– Component 1, 2 and 3)**.

**Fig. 3:**
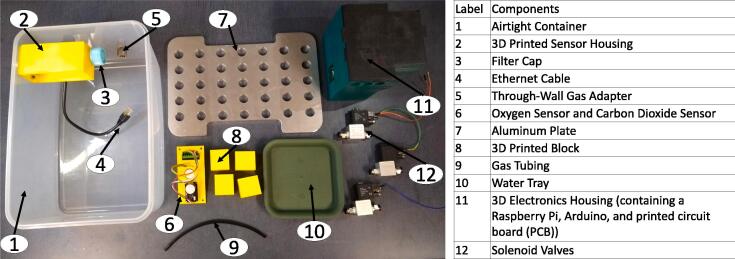
Hypoxia Chamber Macro-Parts.

**Fig. 4:**
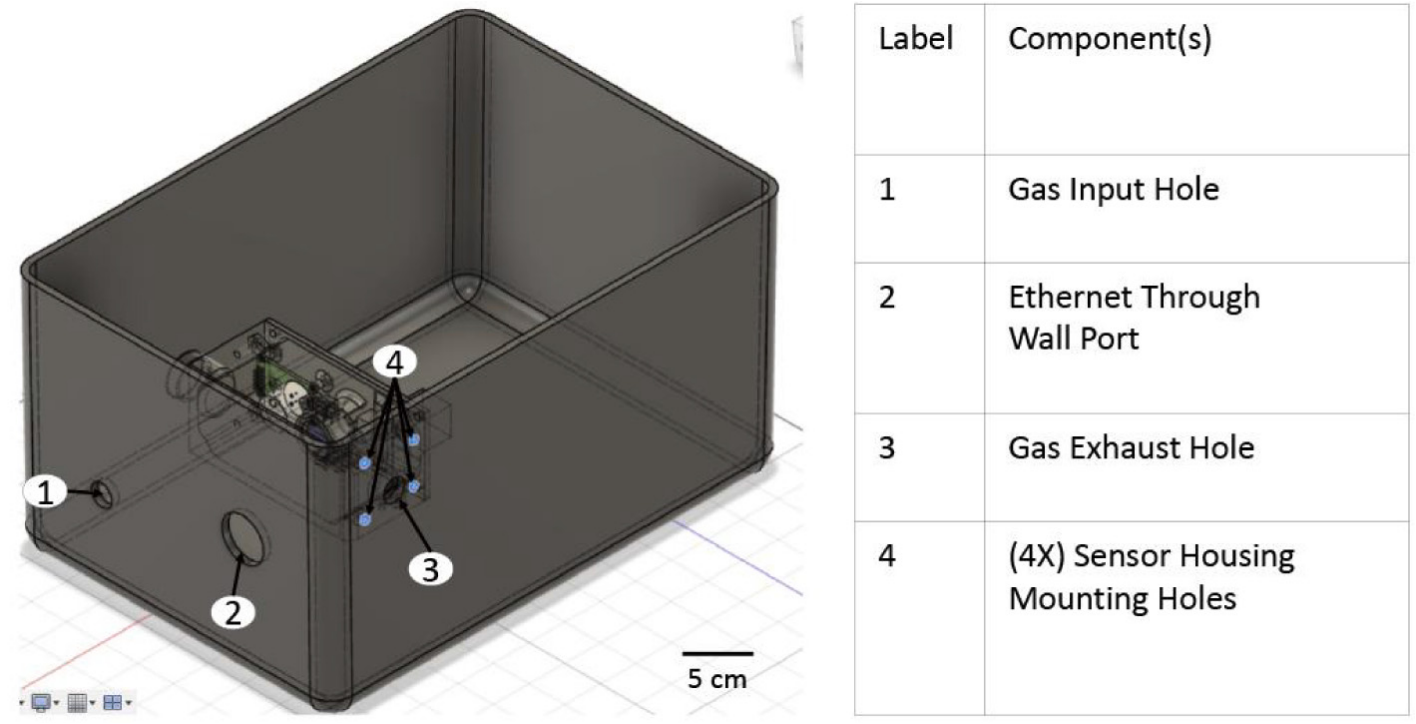
Premade Chamber Model.
  o B) Drill 4 holes in a rectangular pattern around the gas exhaust hole to anchor fasteners for sensor housing (reference *Premade Chamber Full Design* design file for specific hole locations) **(**Fig. 4 **– Component 4)**.
  o *Use standard safety precautions while using a drill (safety glasses, closed-toe shoes, etc.) and secure the chamber using appropriate clamps.
• 5.1.2) 1x Shelf
  o A) Mill out the shelf from stock aluminum (reference *Pre-Made Chamber Shelf* file) **(**Fig. 3**– Component 7)**.* Use standard safety precautions while using a mill
    (safety glasses, closed-toe shoes, etc.) and securely clamp the aluminum in the workspace.
• 5.1.3) 4x Shelf supports
  o A) Using the *Humidity_contol_support* file, print the shelf supports using PLA filament (specifically, we used Sindoh 3DWOX Refill Filament PLA) and a 3D printer. Any printer that can print PLA should suffice **(**Fig. 3**– Component 8)**.
• 5.1.4) Place the shelf supports in the corners of the chamber (not fixed/fastened to the bottom).
• 5.1.5) Fill the water tray with distilled sterile water and set it in the middle of the chamber **(**Fig. 3**– Component 10)**.
• 5.1.6) Place the shelf on top of the supports (the water tray should sit directly below the shelf).
• 5.1.7) Insert the quick-connect fittings into the gas input and exhaust holes and use O-rings to seal holes **(**Fig. 4**– Component 1 and 3)**.
• 5.1.8) Insert the Ethernet through-wall connector into its hole and use an O-ring to seal the hole **(**Fig. 4**– Component 2)**.

### Sensor housing construction

**Fig. 5:**
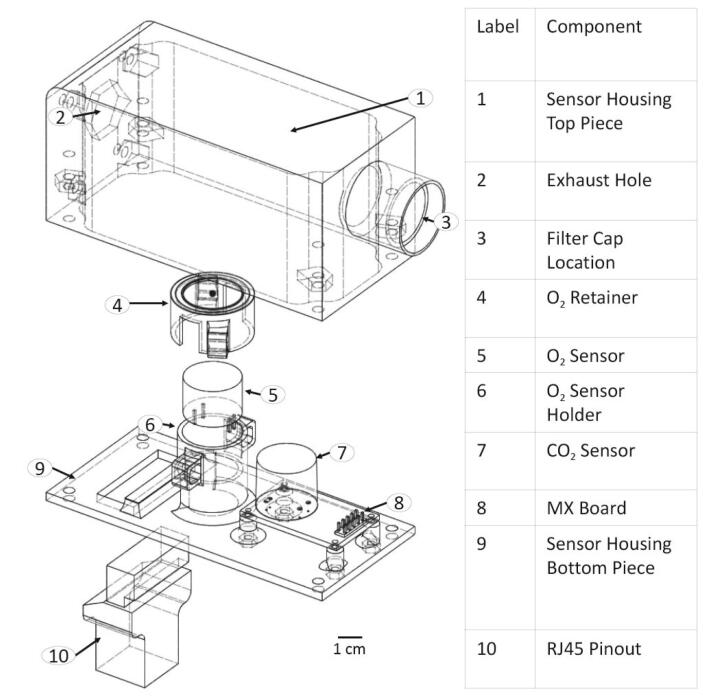
Sensor Housing Schematic.
• 5.2.2) Ensure all pieces fit properly together and that the sensors fit into their positions within in the bottom portion of the sensor housing **(**Fig. 5**)**.
• 5.2.3) Attach the filter cap **(**Fig. 5**– Component 3)**.
• 5.2.4) Mount the top of the Sensor Housing to the chamber wall using 4x 6–32 bolts and nuts with O-rings to seal the holes (see 5.1.1 B for hole references).

• 5.2.1) Using the *Sensor Housing Bottom*, *Sensor Housing Top*, and *O2Sensor Retainer* files, 3D-print both parts of the sensor housing using PLA filament (specifically, we used Sindoh 3DWOX Refill Filament PLA) **(**Fig. 5**– Component 1, 4 and 9)**.

### Electronic housing construction

• 5.3.1) Using the *Electronic Housing* and *Electronic Housing Lid* design files, 3D print the electronic housing and the lid using PLA filament (specifically, we used Sindoh 3DWOX Refill Filament PLA).
• 5.3.2) Mount the Arduino using 4X 6–32 bolts and nuts and mount the Raspberry Pi using 4X 6–32 bolts and nuts.

### Control system assembly

**Fig. 6:**
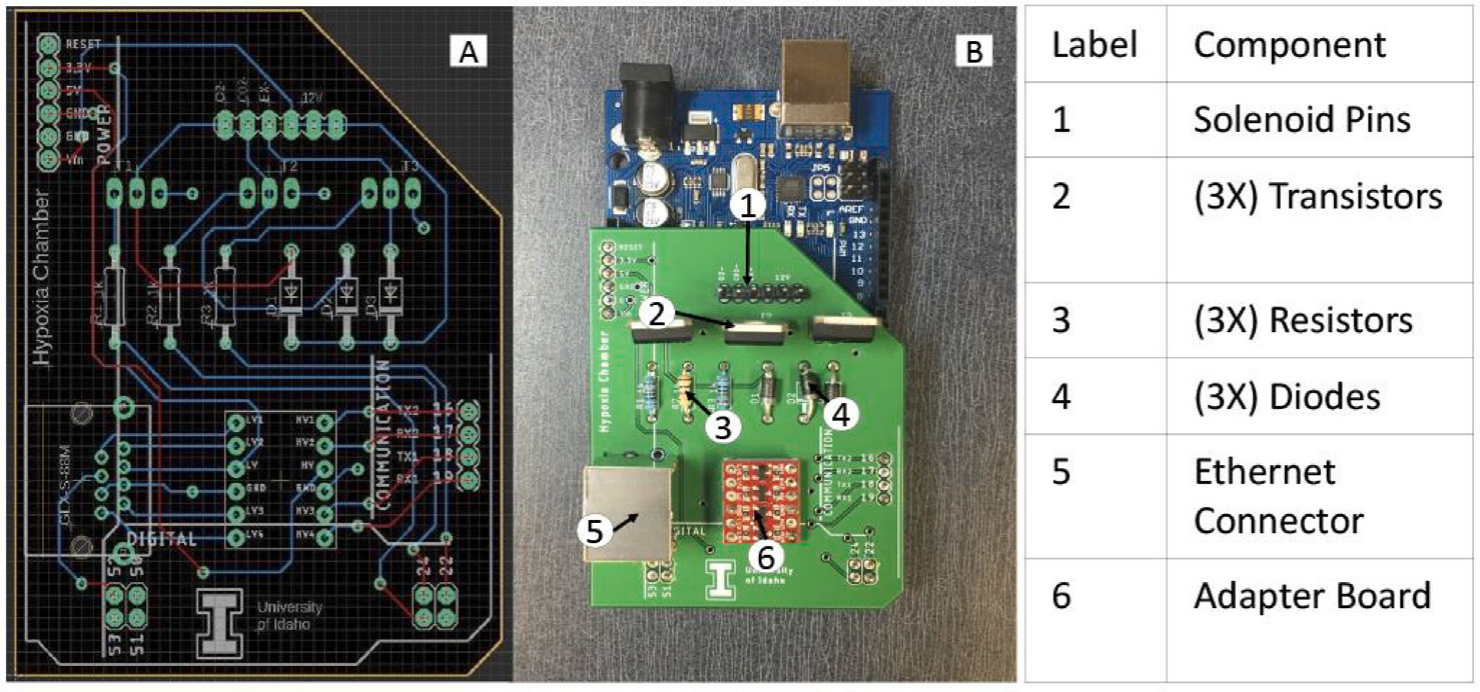
A) PCB Schematic B) PCB Mounted on the Arduino.
• *Safety Hazard* The soldering iron will be extremely hot when soldering. Ensure that the recommended temperature for the desired solder material is used and avoid touching the tip of the iron to skin, hair, clothes, etc., to avoid severe burns. Use proper clamps to hold the PCB while soldering to avoid burns from the heat being conducted through the board.
  o A) Solder:
    ▪ Pin headers (all vias labeled for Arduino, i.e., pins 16/17/18/19/22/24/25/52/Vin/3.3 V/5V/GND) **(**Fig. 6**)**.
    ▪ Negative side of Diode 1 **(**Fig. 6**)**.
    ▪ Middle pin of Transistor 1 (needs top and bottom solder) **(**Fig. 6**)**.
    ▪ 3 1kΩ resistors **(**Fig. 6**)**.
    ▪ 3 1 N004 diodes (facing indicated direction) (except for negative side of D1) **(**Fig. 6**)**.
    ▪ 3 TIP122 Darlington transistors (facing same direction as diodes) (middle pin of T1 needs to be soldered) **(**Fig. 6**)**.
    ▪ Logic Level Converter Board **(**Fig. 6**)**.
    ▪ Ethernet Connector **(**Fig. 6**)**.
    ▪ Pin headers (6 labeled for Solenoids, i.e. O2–, CO2–, EX-, 12 V) **(**Fig. 6**)**.
• 5.4.2) Snap PCB down onto Arduino.
• 5.4.3) Set up solenoids:
  o A) Ensure the solenoids are sufficiently tightened (hand-tight with an adjustable wrench) where tubing connects to the solenoid to prevent gas leaks.
  o B) Place solenoids into specified locations in Electronic Housing **(**Fig. 7 **–Component 1, 2, and 3)**.

• 5.4.1) Once the PCB **(**Fig. 6**)** has been manufactured, solder all the components.

**Fig. 7:**
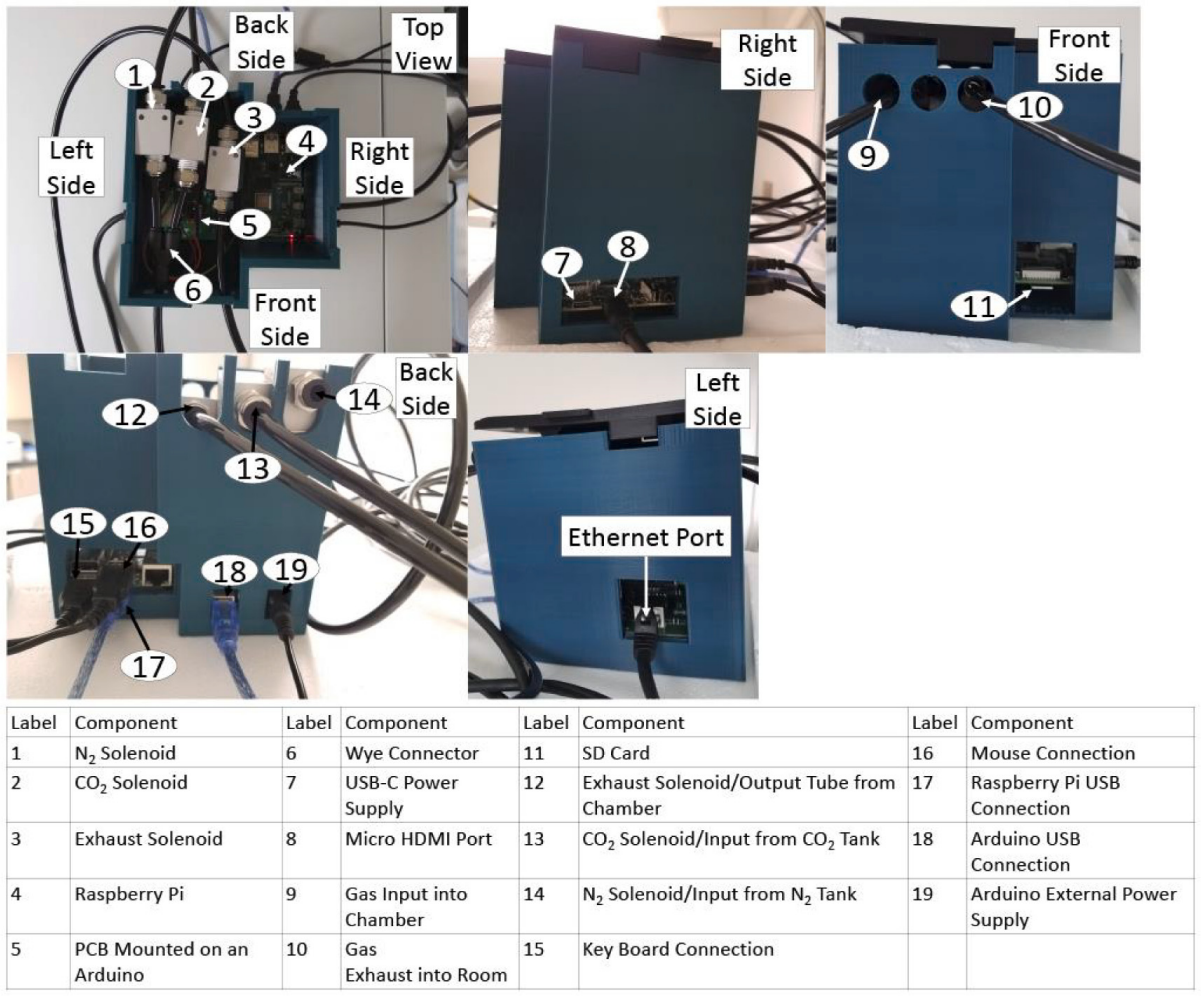
Electronic Housing Unit.
  o C) Attach female-ended wires to solenoid pin headers on PCB, then attach the male ends to the proper solenoids. The orientation of ground and signal wires can be reversed on the solenoid.
  o D) Attach tubing to input and exhaust ports of chamber **(**Fig. 4 **– Component 1 and 3)**.
  o E) Attach CO_2_, N_2_, and chamber exhaust lines into the input valves of the CO_2_, O_2_, and exhaust solenoids, respectively **(**Fig. 7 **– Component 12, 13, and 14)**.
    *Note we refer to the solenoid that is connected to the N_2_ tubing as the O_2_ solenoid due to how the solenoid is referenced in the code.
  o F) Attach tubing to the exhaust valves of the CO_2_, O_2_, and exhaust solenoids, respectively **(**Fig. 7 **– Component 1, 2, and 3)**.
  o G) Attach the chamber input gas tube to the output of wye-connector **(**Fig. 7 **– Component 6 and 9)**.
  o H) Attach CO_2_ and O_2_ solenoid output tubing to the inputs of the wye-connector **(**Fig. 7 **– Component 1, 2, and 6)**.
  o I) Ensure output tube from exhaust solenoid exits Electronic Housing **(**Fig. 7 **– Component 3 and 10)**.
    *Potential air leaks can be tested using the process described in section 7.1 and should only be done in a well-ventilated space using either air or N_2_.
• 5.4.4) Setting up the sensors:
  o A) Attach the Ethernet cable to connector on PCB **(**Fig. 7 **– Ethernet Port)**.
  o B) Attach other end of Ethernet cable to through-wall connector on chamber **(**Fig. 4 **– Component 2)**.
  o C) Epoxy the Ethernet Connector into the sensor housing base so that the screws of the connector are facing away from the O_2_ sensor. Hook the removable wire terminals over the ledge of the sensor housing base.
  o D) Attach male-to-female wires to Ethernet break-out pins, securing them in place by screwing them in.
  o E) Attach the other (female) ends of these wires to the correct sensor pins for the O_2_ and CO_2_ sensors **(**Fig. 8**)**. When connecting the O_2_ sensor, pass the wires through the O_2_ sensor holder.

**Fig. 8:**
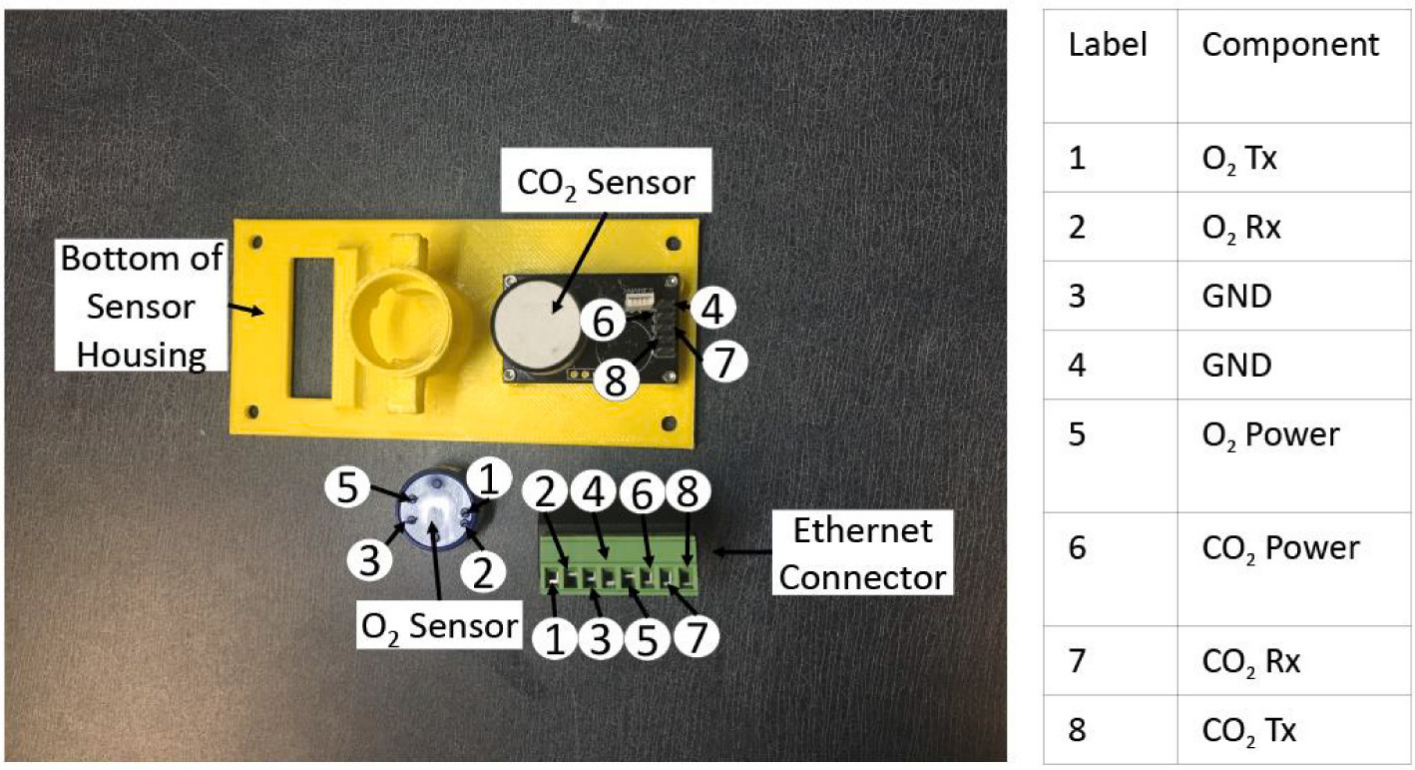
CO_2_ and O_2_ sensors pictured with Ethernet Connector and bottom half of sensor housing.
  o F) Secure the CO_2_ sensor, which should come attached to the *MXBoard*, into the bottom of the sensor housing using the M1.6 bolts and nuts **(**Fig. 5**– Component 7 and 8)**.
  o G) Place the O_2_ sensor into its sensor holder and cover with the O_2_ Sensor retainer **(**Fig. 5**– Component 4, 5, and 6)**.
  o H) Zip-Tie the sensor retainer on.
  o I) Connect Ethernet break-out pins to through-wall ethernet cable inside chamber **(**Fig. 3**– Component 4)**.
  o J) Mount bottom half of sensor housing to top half using 4x 6–32 bolts and nuts inserted into their nut holes **(**Fig. 5**– Component 1 and 9)**.
• 5.4.5) Ensure chamber is securely closed and all necessary components are inside.
• 5.4.6) Set up Arduino and Raspberry Pi:
  o A) Connect both Arduino and Raspberry Pi to their respective power supplies **(**Fig. 7**– Component 7 and 19)**.
    ▪ 120 V AC to 12 V 3A power supply with a barrel plug for the Arduino **(**Fig. 7**– Component 19)**.
    ▪ 120 V AC to 5.1 V 3A USB-C power supply for the Raspberry Pi **(**Fig. 7 **– Component 7)**.
    ▪ *Electric shock hazard – unplug from power until all hardware in secured and covers on the Electronics and Sensor housing are installed.
  o B) Connect Arduino to computer via USB connection.
  o C) In the Arduino IDE install the PID library.
    ▪ After Arduino IDE is installed click on **‘Tools’.**
    ▪ Click on **‘Manage Libraries**…**’**.
    ▪ In the search bar type **PID by b** (the PID library should be one of the first libraries).
    ▪ On the PID library press **‘Install’** (for this project the version that was used was version 1.2.0).
  o D) Upload the Arduino program, *HypoxiaChamber* and *ControlFunctions*, and disconnect the Arduino from the computer.
  o E) Connect Arduino to Raspberry Pi via USB connection **(**Fig. 7 **– Component 17 and 18)**.
  o F) Attach SD card, loaded with Raspbian or desired Linux environment, to Raspberry Pi **(**Fig. 7 **– Component 11)**.
  o G) Connect Raspberry Pi to monitor using proper cables (micro-HDMI to HDMI adapter, then HDMI to VGA cable) **(**Fig. 7 **– Component 8)**.
  o H) Connect Raspberry Pi to a mouse and keyboard **(**Fig. 7 **– Component 15 and 16)**.
  o I) Download or move the *GUI.py* and *settings.csv* files onto a USB or other storage device.
  o J) Connect the storage device to the Raspberry Pi through one of the USB ports.
  o K) Use monitor and keyboard/mouse to copy and paste the *GUI.py* and the *settings.csv* files from the storage device into the Raspberry Pi’s file library.
  o L) GUI can be run using Python3 on the Raspberry Pi.

## Operation instructions

### User interface operation

**Fig. 9:**
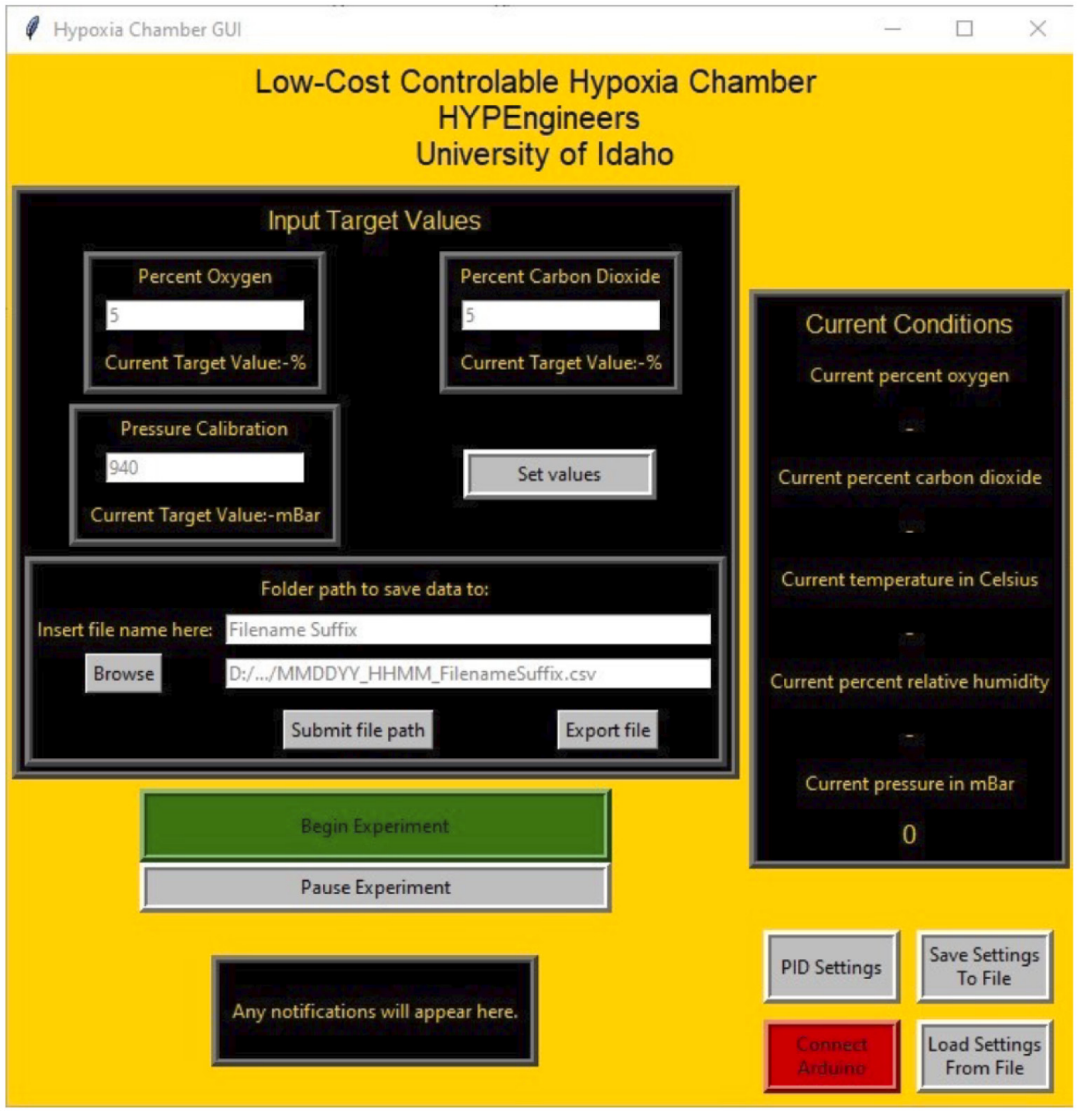
The Graphical User Interface (GUI).
  o B) The Arduino must be connected prior to setting values or beginning an experiment.
• 6.1.2) Setting Values:
  o A) In the appropriate entries, enter the desired CO_2_ and O_2_ percentages that the chamber will maintain **(**Fig. 9**)**.
  o B) Press the **‘Set Values’** button to submit these values. The system should display a notification indicating that it received these values. If no new values are set, the system will **default to run at 5% CO_2_ and 1% O_2_.**
  o C) Set a pressure limit value, this will be used to provide notifications if the chamber pressure exceeds the value.
  o *If pressure limit is exceeded press the **‘Pause Experiment’** button or **‘Press to end Experiment’** button and proceed to open the chamber and let the chamber reach a lower pressure before resuming or restarting an experiment.
• 6.1.3) Saving Files:
  o A) In the filename box insert the suffix to be used for the filename **(**Fig. 9**)**.
  o B) Then browse for a directory that the file can be stored in. A file will be created with a timestamp and then the suffix provided within that directory. (e.g.: “D:/…/MMDDYY_HHMM_FilenameSuffix.csv”)
  o C) Once the file path has been browsed and inserted, press **‘submit file path’** to save it.
  o D) The **‘Export file’** button can be used to copy the experimental file to another directory such as a USB drive. When selected, it will prompt for a new directory path and then copy the current working file to that directory.
• 6.1.4) Running/Stopping an Experiment:
  o A) Connect the CO_2_ and N_2_ gas cylinders to the tubes connected to the CO_2_ and N_2_ solenoids **(**Fig. 7 **– Component 13 and 14)**.
  o B) Set the pressure regulators on the cylinders to about 3–5 PSI.
  o C) Open the gas cylinders.
    *Gas cylinders should always be secured to prevent tip-over.
  o D) Once a save file has been entered and the parameters have been set, press **‘Begin Experiment’** to start. This indicates that gas will begin flowing into the chamber as the system works to reach the desired setpoints, and that all data will be logged in the file specified.
  o E) Select the **‘Press to end Experiment’** button when the experiment has reached its conclusion. This stops the solenoids in the closed position, prohibiting any gas from flowing into the chamber, and it tells the Raspberry Pi to stop saving data in the specified file. The chamber is now safe to open, and all components can be dismantled and sterilized, if necessary.
• 6.1.5) Pausing the Experiment:
  o A) To open the lid and handle the samples during an experiment, the gas flow must be turned off to prevent excess gas leaking into the room. To do so, press the **‘Pause Experiment’** button. This stops the solenoids in the closed position, prohibiting any gas from flowing, but continues to record data measurements in the file specified.
  o B) Once the chamber has been resealed, press the button labeled **‘Experiment paused. Press to resume’** button to resume gas flow.
• 6.1.6) PID Settings:
  o A) Pressing the **‘PID Settings’** button will open another window where the control algorithm can be modified. The proportional (Kp) and integral (Ki) constants that control the PID loops for both the O_2_ and CO_2_ setpoints can be modified here, along with modifying how often the system asks the sensors for measurements and runs the PID loops. If none of these settings are modified, the gray placeholder text indicates the presets that the system will use. The default PID equations are:

• 6.1.1) Connect Arduino:
  o A) The **‘Connect Arduino’** button in the bottom right will be red if the Arduino has not been connected to the Raspberry Pi and GUI **(**Fig. 9**)**. When the Arduino is plugged in via USB to the Raspberry Pi **(**Fig. 7 **– Component 17 and 18)**, press this button to begin serial communication. If properly connected it will turn green and display **‘Arduino Connected’**. If the Arduino is connected on startup, it will automatically connect and turn green.

$$K_{p}e+K_{d}\frac{\mathrm{de}}{\mathrm{dt}}+K_{i}\int_{0}^{t}e\left(t\right)dt$$

$$O_{2}:140e+5\frac{\mathrm{de}}{\mathrm{dt}}+0\int_{0}^{t}e\left(t\right)dt$$

$${\mathrm{CO}}_{2}:150e+5\frac{\mathrm{de}}{\mathrm{dt}}+0\int_{0}^{t}e\left(t\right)dt$$

B) Modified values can be submitted by pressing the **‘Set Values’** button.

• 6.1.7) Default Settings:
  o A) To save set values and PID settings as defaults to be automatically uploaded during future experiments, select the **'Save Settings To File’** button. This saves all of the current settings to the *settings.csv* file in the same directory as the GUI code.
  o B) Upon startup, the GUI checks this file and fills the default values. The values from the file can also be manually loaded using the **‘Load Settings From File’** button.
• 6.1.8) Notification:
  o A) A set of notifications will appear under certain conditions, the reason for the notification and text displayed are listed below:
  o B) Scenario: Default.
• Read-out: **‘Any notifications will appear here.’ (**Fig. 9**)**.
  o C) Scenario: Oxygen value input out of bounds.
    ▪ Read-out: **'Oxygen value too high!'**
    ▪ Meaning: Oxygen value is limited to below 21%.
  o D) Scenario: Carbon Dioxide value input out of bounds.
    ▪ **'Carbon dioxide value too high!'**
    ▪ Meaning: Carbon dioxide value is limited to below 100%.
  o E) Scenario: Target values saved.
    ▪ Read-out: **‘Target gas values accepted. Press 'Begin Experiment' to start.’**
    ▪ Meaning: Target values are within the systems ranges.
  o F) Scenario: Pressure exceeds Setpoint.
    ▪ Read-out: **‘Caution: Pressure exceeds set value! Exercise caution!’**
    ▪ Meaning: Pressure within the chamber has exceed the set values.
  o G) Scenario: Experiment paused.
    ▪ Read-out: **'Program is paused. Door may be opened.'**
  o H) Scenario: Experiment running with no errors.
    ▪ Read-out: **'Do not forget to pause the program to open the door.'**

### Disinfection of chamber/components

• 6.2.1) Partial Disinfection: *
  o A) Remove chamber lid.
  o B) Unplug the ethernet connector connecting the chamber to the electronic housing **(**Fig. 4 **– Component 2)**.
  o C) Disconnect the gas input and exhaust tubing **(**Fig. 4 **– Component 1 and 3)**.
  o D) Remove the shelf from the chamber **(**Fig. 3 **– Component 7)**.
  o E) Remove the shelf supports **(**Fig. 3 **– Component 8)**.
  o F) Remove the water tray and pour out remaining contents **(**Fig. 3 **– Component 10)**.
  o G) Remove filter cap **(**Fig. 3 **– Component 3)**.
  o H) Using a 70% ethanol solution, thoroughly spray or wipe down all parts of the chamber and the components which were removed, this includes the lid of the chamber. Be careful to only wipe down the outside of the sensor housing and the outside of the ethernet cable to avoid damaging the sensors and cable. Allow all pieces to dry before reassembly.
• 6.2.2) Full Disinfection: *
  o A) Remove chamber lid.
  o B) Unplug the ethernet cable connecting the chamber to the electronic housing **(**Fig. 4 **– Component 2)**.
  o C) Disconnect the ethernet cable from the through-wall connector and the sensor housing unit **(**Fig. 3 **– Component 5)**.
  o D) Disconnect the gas input and exhaust tubing **(**Fig. 4 **– Component 1 and 3)**.
  o E) Unmount the sensor housing unit **(**Fig. 3 **– Component 2)**.
  o F) Disconnect the input and exhaust quick-connect fittings **(**Fig. 4 **– Component 1 and 3)**.
  o G) Completely remove the ethernet through-wall connector **(**Fig. 4 **– Component 2)**.
  o H) Remove the shelf **(**Fig. 3 **– Component 7)**.
  o I) Remove the shelf supports **(**Fig. 3 **– Component 8)**.
  o J) Remove the water tray and pour out remaining contents **(**Fig. 3 **– Component 10)**.
  o K) Make sure all O-rings and nuts/bolts have been removed from the chamber.
  o L) Remove filter cap **(**Fig. 3 **– Component 3)**.
  o M) Then follow steps described in 6.2.1) G.

*During disinfection place all parts on a surface that has already been disinfected.

### Reassembly of chamber/components

• 6.3.1) After Partial Disinfection:
  o A) Replace the filter cap with a new sterile one **(**Fig. 3 **– Component 3)**.
  o B) Place chamber into incubator.
  o C) Fill water tray with sterile water slightly below maximum capacity **(**Fig. 3 **– Component 10)**.
  o D) Place the water tray back into the chamber with sterile water.
  o E) Place the shelf supports into the chamber **(**Fig. 3 **– Component 8)**.
  o F) Place the shelf onto the humidity shelf supports **(**Fig. 3 **– Component 7)**.
  o G) Pass the tubes and cables through the port in the incubator.
  o H) Reseal the port with a stopper and further cover the hole with parafilm.
  o I) Reconnect the gas input and exhaust tubbing **(**Fig. 4 **– Component 1 and 3)**.
  o J) Reconnect the ethernet cable from the electronic housing unit **(**Fig. 4 **– Component 2)**.
• 6.3.2) After Full Disinfection
  o A) Follow steps 5.1.4–5.1.8.
  o B) Follow steps 5.2.3–5.2.4.
  o C) Before placing the water tray in the chamber, place the chamber in the incubator.
  o D) Pass the tubes and cables through the port in the incubator.
  o E) Reseal the port with a stopper and further cover the hole with parafilm.
  o F) Reconnect the ethernet cable from the electronic housing unit to the chamber **(**Fig. 4 **– Component 2)**.
  o G) Reconnect the ethernet cable from the through wall ethernet connector to the sensor housing unit **(**Fig. 3 **– Component 5)**.
  o H) Reconnect both the input and exhaust tubes **(**Fig. 4 **– Component 1 and 3)**.

### Preparation for an experiment

• 6.4.1) Before beginning an experiment, it is recommended to have the chamber sit inside the incubator for an appropriate amount of time to acclimate (generally, at least one hour). This allows the chamber to acclimate to the temperature, humidity, and CO_2_ levels of the incubator. Therefore, after sterilizing the chamber and its components (i.e., the shelf, shelf supports, water tray, sensor housing), set the chamber inside the incubator with the lid off. Leave the chamber to reach the incubator’s desired conditions, then place the experimental material (e.g., stem cells in culture plates) inside the chamber, and seal the chamber with the latches on the lid. The experiment can now be started by using the GUI as described above. It is also recommended that the entire system be disinfected after each experiment as described in 6.2.1 and 6.3.1 to limit the potential for contamination. After about 3 to 4 experiments, it is recommended to conduct a full disinfection as described in 6.2.2 and 6.3.2.

## Validation and characterization

### Airtight

• 7.1.1) The airtightness of the chamber was tested via placing soapy water around the edges and the holes of the chamber and pumping air into the chamber. No bubbles were formed in these locations, indicating the chamber was airtight. Airtightness is important for this system to reduce N_2_ and CO_2_ gas usage. However, airtightness is not absolutely critical to the overall functionality of the system. It is more important that there is minimal pressure build-up within the system, which was evaluated in section 7.2.

### Validating pressurization and equilibrium

**Fig. 10:**
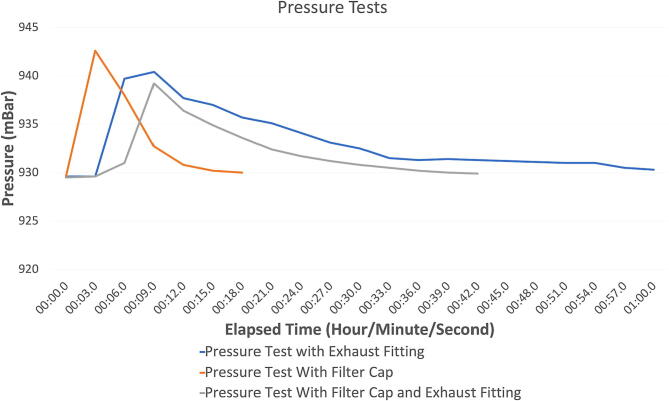
Chamber Pressure Test.

• 7.2.1) A bike pump was connected to the chamber and all ports, excluding the exhaust port, were blocked. The chamber was filled with three pumps of air via the bike pump, and the pressure was continuously recorded until the chamber dropped back to the starting pressure. The edges of the chamber were covered in soapy water to test if the pressure compromised the airtight seal. This assessment was conducted first with only the exhaust fitting, then with only the filter, and finally with both. The results of each test are shown in Fig. 10.

**Fig. 11:**
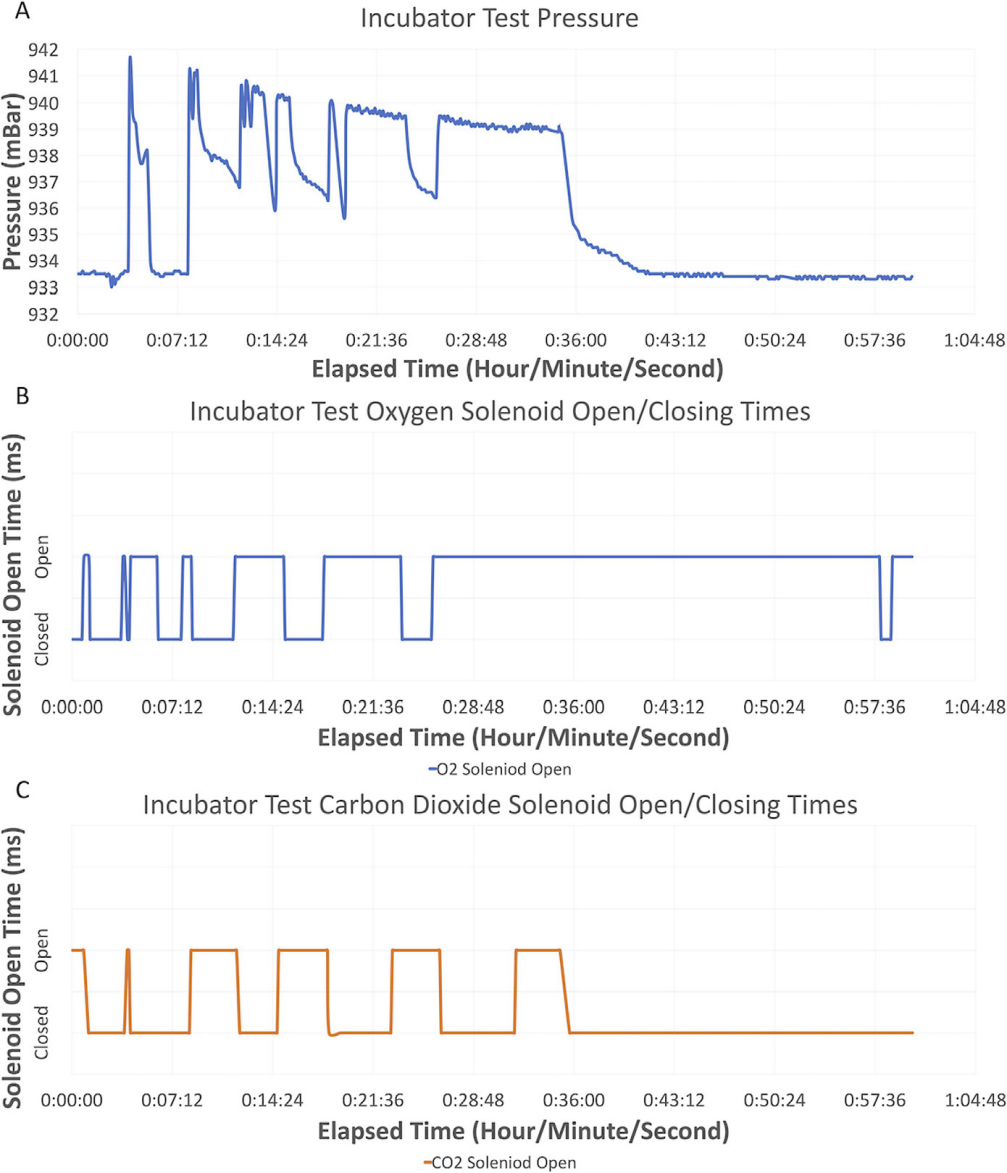
A) The pressure readings within the chamber while the system was in use within the incubator. B) The opening and closing times of the oxygen solenoid. C) The opening and closing times of the carbon dioxide solenoid.

In all three cases, in Fig. 10, the pressure reached equilibrium in under 1 min. The chamber was able to withstand a pressure increase of roughly 12.5 mBar without forming any bubbles, hence not compromising the airtight seal. As described further in Fig. 11 when N_2_ gas is input using a tank regulator pressure of around 3 psi, while having the exhaust system functioning, the slightly increased pressure does not result in leaks or breaches of the chamber integrity. We also tracked the pressure in the chamber while performing a test of the entire system, which included inputting both N_2_ and CO_2_ and exhausting the gas in the chamber until specific O_2_ and CO_2_ set points were achieved. The results of this test are shown in Fig. 11.

Based on these results, the hypoxia chamber system can withstand the pressure produced through inputting gases. This also shows that once gases reached their set points and the input of gasses stopped (occurring at 36 min) the pressure achieved equilibrium around 7 min later. Note: The jagged peaks in Fig. 11A are indicative of each time gas was injected into the chamber (Fig. 11B, C).

### Maintaining O_2_ and CO_2_ levels

**Fig. 12:**
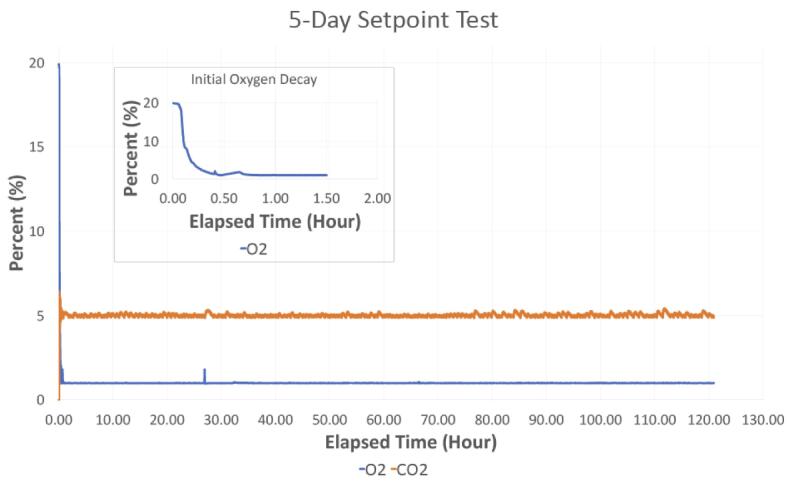
5-day chamber test with a 5% CO_2_ and 1% O_2_ setpoint. The inset shows the initial oxygen decay.

• 7.3.1) To validate that our chamber can maintain the desired O_2_ and CO_2_ setpoints for an extended period of time, the chamber was monitored over the course of 5 days while at room temperature. The setpoints used for this test were 5% CO_2_ and 1% O_2_. The results of this test (shown in Fig. 12), demonstrates the system successfully reached and maintained the desired setpoints for the duration of the test period with minimal fluctuations in the gas levels.

**Fig. 13:**
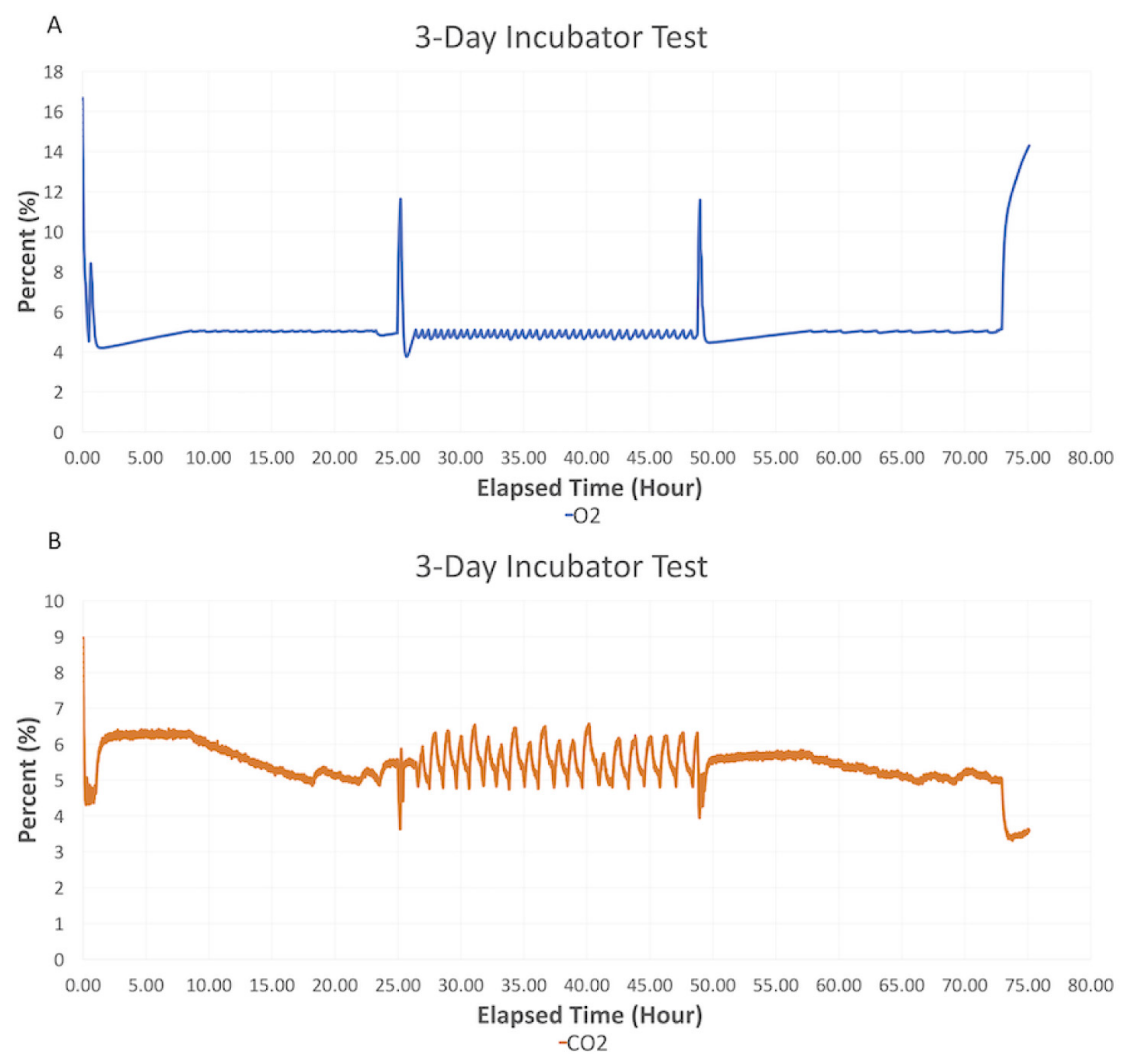
A) Oxygen and B) carbon dioxide readings during the 3-day incubator test with two 12-well plates of mesenchymal stem cells (Large peaks correspond to times when the chamber was opened, and oscillations shown between 25 h and 50 h are due to the lid of the chamber not being completely closed by the user).

**Fig. 14:**
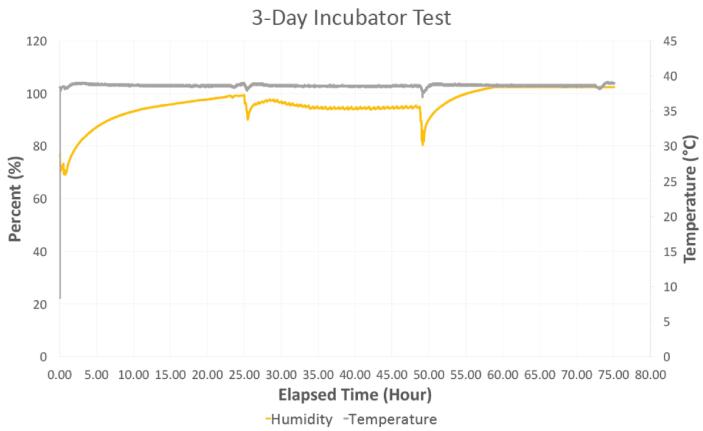
Relative humidity and temperature during a 3-day incubator test with two 12-well plates of mesenchymal stem cells.

A subsequent experimental test using live cells that were cultured for three days inside the hypoxia chamber was also conducted. Prior to beginning the experiment, the chamber was placed inside the incubator with its lid partially open (i.e., one side was left unhinged) for 24 h to acclimate to incubator conditions. After 24 h, two 12-well plates of a mouse mesenchymal stem cell line (C3H10T1/2, American Tissue and Cell Collection, Manassas, VA) were placed inside the chamber, and it was fully sealed. The setpoint values for the experiment were 5% CO_2_ and 5% O_2_. The chamber was then opened once per day for observation and/or culture medium supplementation over the next three days. These events correspond to peaks in Fig. 13 and Fig. 14.

As seen in Fig. 13, between 25 and 50 h, the CO_2_ and O_2_ readings fluctuated minimally. The fluctuations began when the chamber was opened on day 2 and ended when it was opened on day 3. It was noted that the lid of the chamber had not been completely sealed after opening it on day 2 causing it to leak. However, even with a poor seal, the system appeared robust and was able to maintain an environment between 4 and 6% CO_2_ and 4–5% O_2_, which was within the desired environmental conditions for this experiment.

For each of these tests within the cell culture incubator, the CO_2_ concentration was initially benchmarked against the 5% CO_2_ of the incubator. Further, the cell culture medium contains a pH indicator (phenol red), which appeared similar to controls grown in a standard cell culture incubator containing 5% CO_2._ Together, this helped to demonstrate the system’s ability to track and maintain CO_2_ in the chamber.

### Limitations

• 7.4.1) While this system successfully meets the requirements for functionality and maintaining the appropriate hypoxic environment, there are still limitations to the current design. One such limitation is not having a controllable time for reaching the desired setpoints. Currently, the system has a set time to reach O_2_ and CO_2_ setpoints within approximately 30 min. However, this can be tuned using the PID controller and may need to be customized for each application.
• 7.4.2) There is no direct control over the temperature or humidity within the chamber, only passive control through the ambient temperature and the water tray. Thus, for mammalian cell culture, this hypoxia chamber cannot be used independently of a cell culture incubator.
• 7.4.3) The CO_2_ sensor was benchmarked against the 5% CO_2_ concentration of the cell culture incubator. Similarly, the O_2_ sensor was evaluated by benchmarking it against atmospheric levels of O_2,_ and when it was placed inside a closed container with a lit candle. Initially the O_2_ concentration was at atmospheric levels, but as the candle burned O_2_ concentration approached zero until the candle was extinguished. A more rigorous multi-point calibration could be warranted.

## Recommended changes/future work

### Recommended changes

**Fig. 15:**
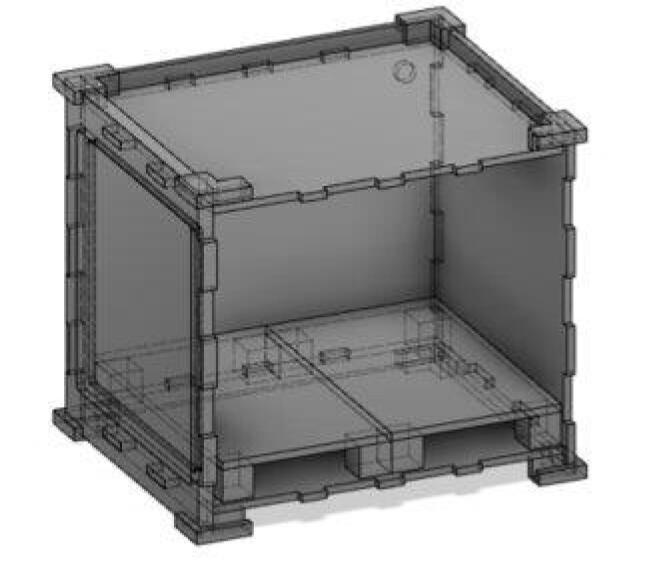
Customizable chamber design.

The initial design of the hypoxia chamber used laser-cut acrylic sheets that could be assembled to create a custom chamber **(**Fig. 15**)**. The acrylic sheets were to be held together using a series of dovetail joints sealed with Epoxy and 3D printed corner pieces for stability. A single modified sheet of acrylic could then be combined with an O-ring and external hinges to create an airtight door. Internal chamber housing (e.g., shelves, water tray, electronic) and gas/wiring inputs/outputs would be in the same locations as our final design, as outlined above. The Acrylic was initially selected for its transparency and affordability. Unfortunately, prototype testing found that laser-cut acrylic edges are prone to crazing when in contact with ethanol (which is commonly used for sterilization), compromising the material. However, a different material, such as polycarbonate, could potentially be used in place of acrylic with minimal modifications to the design to resolve these issues. As such, the initial design files can be found in **‘Custom Chamber’** section for future use and/or modification. Additionally, to evaluate the configuration of a custom chamber design it may be helpful to simulate gas flow (see Supplemental Materials and Fig. S1 for an example). The efficacy of polycarbonate was not assessed because it did not fulfill our design criteria, namely affordability and ease-of-access (i.e., polycarbonate requires milling vs laser-cutting). Thus, the idea to use a pre-made plastic container was pursued for the final design.

Helpful modifications to the GUI may include adding real-time graphing and remote connectivity. With the current design, stored CSV files created during use of the chamber can later be collected and uploaded to an appropriate program (such as Microsoft Excel) to generate plots and conduct analysis after the experiment has ended. Though the GUI provides real-time reading, real-time graphing of O_2_ and CO_2_ levels would allow instant troubleshooting and assist in monitoring for potential gas fluctuations throughout the duration of the experiment. Similarly, incorporating remote connectivity with the GUI would benefit efficiency. Remote connectivity could allow users to monitor the system from afar during an experiment. Incorporating remote connectivity with system notifications could, for example, notify if the pressure limits have been exceeded within the chamber or if the system is detecting gas leakage. Potential methods to incorporate this include using a Python program to create a web application that could be accessed remotely or creating an email and/or SMS notification system. Note that both methods require internet connectivity.

Other adjustments include reworking the sensor housing and creating a second PCB for the sensors in order to reduce clutter within the sensor housing. A second PCB would have pin headers in the appropriate places so that the O_2_ sensor could plug in, and the CO_2_ sensor board could be connected via a ribbon cable. These pin headers would then be routed to an ethernet connector like the one on the PCB that was made for the Arduino. This ethernet connector would then exit the wall of the chamber directly, so that the two PCBs would be connected by a single ethernet cable, effectively removing the need for the through-wall ethernet and breakout adapters. However, the sensor housing itself would need to be redone to accommodate the PCB and ensure that the ethernet connector could exit, which would require another hole to be drilled into the chamber. This redesign could minimize in-housing wiring, ensure good connections to the sensors, and securely hold the sensors in place.

It is also possible to change the design so that an Arduino is not necessary. This could have been done by using the Raspberry Pi 4′s ability to support multiple universal asynchronous receiver-transmitter (UART) devices. Using multiple UART devices we could eliminate the need for an Arduino, possibly reducing the cost of the overall system.

Additionally, the shelf could be made from a variety of materials, not just aluminum. In this case, an aluminum shelf was utilized due to ease of access. As long as the material can be sterilized or disinfected, and is compatible with the incubator environment, it can be used in the design (e.g., a 3D printed shelf).

### Future work

Overall, we demonstrated a low-cost, open-source, and controllable hypoxia chamber that can fit inside a standard cell culture incubator. Thus, this system can be easily reproduced in other laboratories to advance future work in tissue engineering and regenerative medicine, novel drug discovery and cancer therapies, and cell and tissue pathogenesis. As the system can easily be customized based on the foundation we provided, it could be modified to fit animal cages for conducting *in vivo* hypoxia studies. Our future research using this hypoxia chamber will be aimed at elucidating the specific downstream targets of HIF-1α for applications in tissue engineering and identifying the impacts of physiological O_2_ levels on other potential signaling pathways and differentiation in stem cells.

## Human and animal rights

No human or animal studies were conducted in this work.

## Declaration of Competing Interest

The authors whose names are listed certify that they have NO affiliations with or involvement in any organization or entity with any financial interest.
