# Supplementary material for: Low-cost, open-source cell culture chamber for regulating physiologic oxygen levels
Source: HardwareX. 2021 Dec 18;11:e00253. doi: 10.1016/j.ohx.2021.e00253 (PMC9058583; doi:10.1016/j.ohx.2021.e00253)
Supplement: Supplementary data 1 [file mmc1.docx]

**Supplemental Material:**

Gas Flow Simulation: To visualize gas circulation within the chamber and evaluate if input gas disperses throughout the chamber or is immediately removed through the exhaust port, a computational fluid dynamics (CFD) model was assessed using Autodesk CFD (Autodesk Inc., San Rafael, CA).  This can be run by downloading the [Premade Chamber Gas Flow Validation](https://osf.io/ntc26/?view_only=efbd17c8ccfe4513b65366ae39c4c2fd) folder. In summary, after unzipping the folder, open the CFD file, **‘Premade_chamber_6_faceup_Simulation'**, in the Autodesk CFD program. Press **‘solve’** in the ribbon, in the pop window, set the iteration ‘to continue from’ to be 0, select the desired number of iterations (we used 50 iterations) and then press solve in the same window. Once complete, traces will appear, which represent the gas flow through the system. To modify the traces of the simulation, go to the results section, select **‘trace’**, in the ‘create set section’ choose **‘circular seed type**,**’ ‘seed pattern grid’**, and choose your desired seed density, in this example a seed density of 0.15 was used (which is based on runtime and resolution). Then select the lowest hole on the body and expand the red dots until they cover the entire circle and then release the left click on the mouse. This should produce results similar to what is shown in**Figure S1**. This simulation showed that input gas appeared to be distributed throughout the chamber.

Note: This simulation only accounts for input of N_2_ gas. The simulation shows that the gas will most likely circulate throughout the center of the chamber before leaving the chamber through the exhaust port. A limitation is that circulation may be reduced at the rectangular corners of the chamber. However, based on this simulation, the primary testing sites (i.e., the central region) of the chamber appear to be uniform.


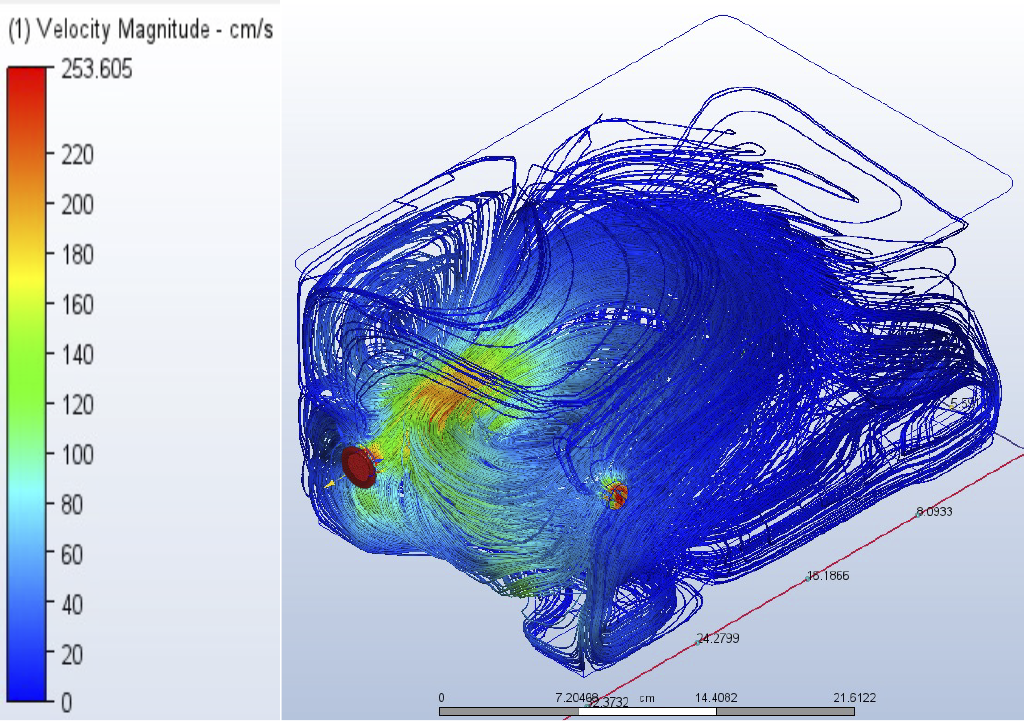


*Figure S1: Gas Flow Simulation*
